# Multi-ancestry genome-wide association analyses provide insights into the genetic basis of Hashimoto’s thyroiditis

**DOI:** 10.1038/s41588-026-02704-w

**Published:** 2026-07-29

**Authors:** Melissa N. Bujnis, Rosalie B. T. M. Sterenborg, Yong Li, Bjørn Olav Åsvold, Luka Brčić, Vesna Boraska Perica, Anav Babbar, Joshua C. Denny, Lars G. Fritsche, Masahiro Kanai, Ilze Konrade, Graham Leese, Eirini Marouli, Andres Metspalu, Marta R. Moksnes, Bhramar Mukherjee, Yukinori Okada, Colin N. A. Palmer, Areti Papadopoulou, Raitis Peculis, Vita Rovite, Peter J. Sauer, Enrique Soto-Pedre, Sundararajan Srinivasan, Inga Steinbrenner, Maris Teder-Laving, Bin Wang, Antoine Weihs, Chenjie Zeng, Jin Zhou, The Biobank Japan Project, Yukinori Okada, XiCheng Song, Lynn B. Jorde, Marco Medici, Alexander Teumer

**Affiliations:** 1 Department of Human Genetics, University of Utah, Salt Lake City, UT USA; 2 Department of Internal Medicine, Division of Endocrinology, Radboud University Medical Center, Nijmegen, The Netherlands; 3 Academic Center for Thyroid Diseases, Department of Internal Medicine, Erasmus Medical Center, Rotterdam, The Netherlands; 4 Institute of Epidemiology and Prevention, Faculty of Medicine and Medical Center - University of Freiburg, Freiburg, Germany; 5 HUNT Center for Molecular and Clinical Epidemiology, Department of Public Health and Nursing, NTNU, Norwegian University of Science and Technology, Trondheim, Norway; 6 Department of Endocrinology, Clinic of Medicine, St. Olavs Hospital, Trondheim University Hospital, Trondheim, Norway; 7 Department of Medical Biology, School of Medicine, University of Split, Split, Croatia; 8 Precision Health Informatics Section, National Human Genome Research Institute, National Institutes of Health, Bethesda, MD USA; 9 All of Us Research Program, Office of the Director, National Institutes of Health, Bethesda, MD USA; 10 Department of Biostatistics, University of Michigan School of Public Health, Ann Arbor, MI USA; 11 Center for Statistical Genetics, University of Michigan School of Public Health, Ann Arbor, MI USA; 12 Analytic and Translational Genetics Unit, Massachusetts General Hospital, Boston, MA USA; 13 Program in Medical and Population Genetics, Broad Institute of MIT and Harvard, Cambridge, MA USA; 14 Department of Statistical Genetics, Osaka University Graduate School of Medicine, Suita, Japan; 15 Department of Internal Medicine, Riga Stradins University, Riga, Latvia; 16 NHS Tayside, Dundee, Scotland UK; 17 William Harvey Research Institute, Barts and The London School of Medicine and Dentistry, Queen Mary University of London, London, UK; 18 Digital Environment Research Institute, Queen Mary University of London, London, UK; 19 Estonian Genome Centre, Institute of Genomics, University of Tartu, Tartu, Estonia; 20 Department of Research, St. Olav University Hospital, Trondheim, Norway; 21 HUNT Research Center, Department of Public Health and Nursing, Faculty of Medicine and Health Sciences, Norwegian University of Science and Technology, Levanger, Norway; 22 Department of Biostatistics, Department of Chronic Disease Epidemiology and Department of Statistics and Data Science, Yale University, New Haven, CT USA; 23 Department of Genome Informatics, Graduate School of Medicine, the University of Tokyo, Tokyo, Japan; 24 Laboratory for Systems Genetics, RIKEN Center for Integrative Medical Sciences, Yokohama, Japan; 25 Laboratory of Statistical Immunology, Immunology Frontier Research Center (WPI-IFReC), Osaka University, Suita, Japan; 26 Division of Population Health & Genomics, School of Medicine, Ninewells Hospital & Medical School, University of Dundee, Dundee, UK; 27 Latvian Biomedical Research and Study Centre, Riga, Latvia; 28 Faculty of Medicine and Life Sciences, University of Latvia, Riga, Latvia; 29 Department of Immunology, Binzhou Medical University, Yantai, China; 30 Department of Psychiatry and Psychotherapy, University Medicine Greifswald, Greifswald, Germany; 31 Department of Endocrinology and Foot & Ankle Surgery, Yantai Yuhuangding Hospital, Qingdao University, Yantai, China; 32 Shandong Provincial Medicine and Health Key Laboratory of Endocrine and Metabolic Disease and Clinical Translational Medicine, Yantai, China; 33 Department of Otorhinolaryngology, Head and Neck Surgery, Yantai Yuhuangding Hospital, Qingdao University, Yantai, China; 34 Shandong Provincial Key Laboratory of Neuroimmune Interaction and Regulation, Yantai, China; 35 DZHK (German Center for Cardiovascular Research), partner site North, Greifswald, Germany; 36 Department of Population Medicine and Lifestyle Diseases Prevention, Medical University of Bialystok, Bialystok, Poland

**Keywords:** Thyroid diseases, Genome-wide association studies

## Abstract

Autoimmune hypothyroidism (Hashimoto’s thyroiditis) is common and has a strong genetic component. Here we performed multi-ancestry genome-wide association meta-analyses encompassing 48,694 Hashimoto’s thyroiditis cases, using a precise case definition, and 1,044,134 controls. We identified 155 significant (*P* < 5 × 10^−8^) independent genetic associations, of which 45 variants and 19 loci were not previously associated with hypothyroidism. Six loci were specific for individuals of European ancestry reference populations. Functional enrichment analyses of Hashimoto’s thyroiditis-associated genes highlighted immune cells and the spleen, underpinning the importance of T cells in Hashimoto’s thyroiditis development. This observation was further supported by 161 significant colocalizations with expression quantitative trait loci in immune cells and 40 in thyroid tissue (for example, *TG, VAV3*, *IRF5*), highlighting the interplay between the immune system and the thyroid. Mendelian randomization indicated causal effects of Hashimoto’s thyroiditis on cardiovascular traits and expected associations with thyroid hormone levels.

## Abstract (web summary)

Multi-ancestry genome-wide association analyses of Hashimoto’s thyroiditis identify new risk loci and highlight shared genetic architecture with other autoimmune diseases and pleiotropic effects on clinical outcomes.

## Main

Autoimmune thyroid diseases are a group of chronic conditions affecting the thyroid gland, of which the most common is autoimmune hypothyroidism; that is, hypothyroidism resulting from Hashimoto’s thyroiditis. Hashimoto’s thyroiditis is a common disease, affecting ~3% of US and European populations^1–4^. In Hashimoto’s thyroiditis, the immune system attacks the thyroid gland, eventually resulting in decreased production of thyroid hormones (hypothyroidism). The thyroid hormones thyroxine (T4) and triiodothyronine (T3) are essential for normal growth and differentiation of cells and tissues, regulation of energy metabolism and development and physiological function of virtually all human tissues^5^. Therefore, patients with hypothyroid Hashimoto’s thyroiditis require lifelong T4 replacement therapy (levothyroxine). However, this treatment does not treat the underlying immune dysregulation that is actively destroying the thyroid^6^. Hashimoto’s thyroiditis has been associated with a wide range of adverse health outcomes, including a higher risk of several comorbidities such as obesity, type 2 diabetes, infertility and pregnancy complications, depression, cognitive dysfunction and cardiovascular diseases, as well as an increased risk of overall mortality^5^. The pathophysiology of Hashimoto’s thyroiditis involves initial damage to thyroid cells followed by subsequent auto-antigen release and presentation^7^. These autoreactive immune cells attack the thyroid, resulting in the activation of immune responses (both cellular and humoral), including cytokine production and cytotoxicity^7^. In the majority of Hashimoto’s thyroiditis cases, antibodies against thyroperoxidase (TPOAb) are detectable in blood^6^. Hashimoto’s thyroiditis development is influenced by a combination of genetic and environmental factors, including sex, age, socioeconomic status and iodine intake^2,3^.

The prevalence of diagnosed overt hypothyroidism (low T4, high thyroid-stimulating hormone (TSH)) in the general population ranges from 0.2% to 5.3% in Europe and 0.3% to 3.7% in the USA^8–11^. Prevalence estimates differ owing to the case definition and the population differences between studies. The NHANES III study, based on census data from the USA, determined that the prevalence of hypothyroidism was similar in white and Hispanic individuals (4.6%) but was significantly lower in individuals of African descent (1.7%)^9^. Additionally, the prevalence of hypothyroidism, specifically Hashimoto’s thyroiditis, is approximately four times greater in females than males^8,9,11,12^. Twin studies have suggested that genetic factors are major determinants in the development of Hashimoto’s thyroiditis, with an estimated heritability of ~65%^13,14^. A substantial part of our current understanding of the genetics of Hashimoto’s thyroiditis comes from genome-wide association studies (GWAS). Previous Hashimoto’s thyroiditis GWAS associations implicated thyroid-specific genes and genes involved in general immune response regulation, T cell regulation, cell-mediated destruction and auto-antigen presentation^15–19^. Although previous GWAS have been productive, their limitations include the use of an overly broad case definition (that is, including non-autoimmune hypothyroidism or other autoimmune thyroid diseases), small sample sizes and focus on single ancestries, predominantly European genetic ancestry^15–19^. Here, we conducted multi-ancestry and sex-stratified GWAS meta-analyses using a precise definition of cases and disease-free controls in ten multi-ancestry cohorts. Having a comparable number of cases compared to previous large autoimmune thyroid disease GWAS, our results yielded novel associations, demonstrating the importance of having a precise case definition for a highly heterogeneous disease.

## Results

### Multi-ancestry GWAS results

**Fig. 1:**
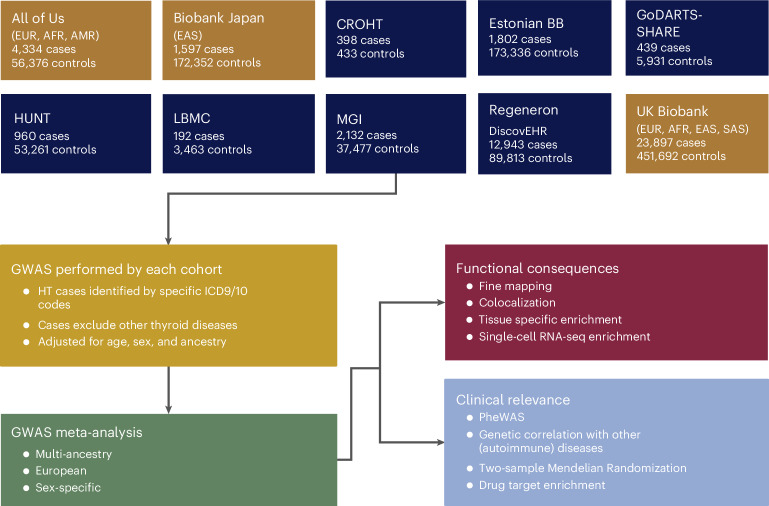
Study design for GWAS meta-analysis of Hashimoto’s thyroiditis. Study design of the Hashimoto’s thyroiditis GWAS meta-analysis including ten multi-ancestry international cohorts (Supplementary Table 1). HT, Hashimoto’s thyroiditis. Cohorts that include or consist of non-European genetic ancestry reference individuals are colored in brown, with the major genetic ancestry reference populations given in brackets (EUR, European; AFR, African; AMR, admixed American; EAS, East Asian; SAS, South Asian). Follow-up analyses were performed using the meta-analysis results to identify the underlying mechanisms of the specific genome-wide variants and the translation to clinical diagnoses.

**Fig. 2:**
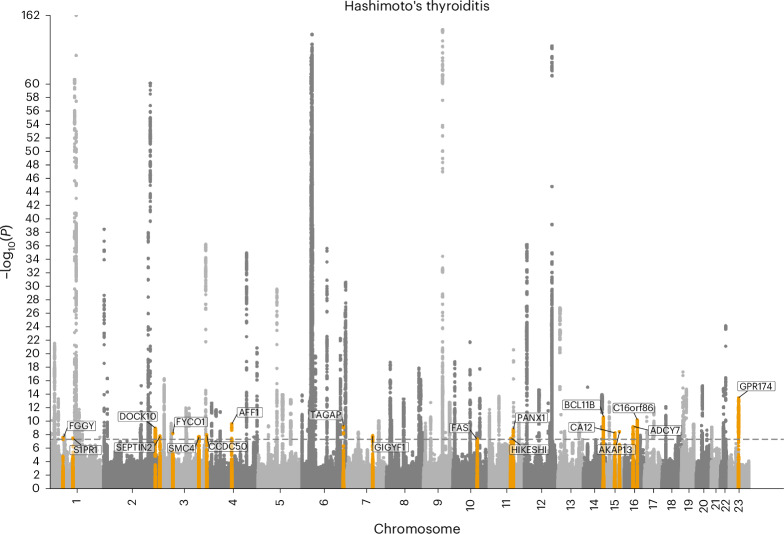
Multi-ancestry genome-wide association results for Hashimoto’s thyroiditis. Variants are plotted on the *x* axis according to their position on each chromosome, with the −log_10_(*P* value) of the association test on the *y* axis. The horizontal line indicates the threshold for genome-wide significance (*P* = 5 × 10^−8^). All *P* values were obtained from two-sided association tests (*z-*statistics); correction for multiple testing is indicated by the level of genome-wide significance. Novel loci are colored in orange and labeled by the annotated gene name.

We conducted a GWAS meta-analysis of Hashimoto’s thyroiditis comprising 48,694 cases and 1,044,134 controls across ten cohorts from Europe, the USA and Japan (Fig. 1 and Supplementary Table 1). The individuals included were predominantly but not exclusively similar to European ancestry reference populations (European, 75%; East Asian, 16%; African, 2%; South Asian and Middle Eastern, 1%; admixed or unknown, 6%) (Supplementary Table 1). Genetically determined larger non-European ancestry populations include individuals similar to African, East and South Asian reference populations in the UK Biobank^20^ and to African and admixed Americans in the ‘*All of Us*’ Research Program. Cases were defined using ICD9/10 codes for autoimmune hypothyroidism and unspecified hypothyroidism, and importantly excluded other thyroid diseases known to cause hypothyroidism or Hashimoto’s thyroiditis, such as post-partum thyroiditis, hyperthyroidism, thyroid cancer and medication-induced hypothyroidism. Individuals without thyroid dysfunction and over the age of 18 years were considered as controls. Details of the case and control definitions are provided in Supplementary Table 2. In total, 8,148,325 variants were analyzed using METAL^21^ after quality control filtering, including a minor allele frequency (MAF) of 1% and high confidence in the imputed allele, as further outlined in the Methods. Overall, 20,996 variants were genome-wide significantly (*P* < 5 × 10^−8^) associated with Hashimoto’s thyroiditis in our study: 155 independent variants in 124 unique loci (Fig. 2 and Supplementary Table 3). Quantile–quantile plots are shown in Supplementary Figure 1. The linkage disequilibrium (LD) score (LDSC) regression intercept (Methods) of 0.97 indicated no inflation of the results by unaccounted population stratification.

In total, we identified 45 novel genetic variants and 19 novel loci (Table 1 and Supplementary Table 3) after comparison with the results of the most recent GWAS of hypothyroidism, which included other causes of hypothyroidism based on levothyroxine medication, such as congenital hypothyroidism and post-thyroid cancer or Graves’ disease treatment^17,22,23^. In comparison, we used a more precise definition of cases by excluding individuals with other thyroid diseases that cause hypothyroidism to best represent autoimmune hypothyroidism/Hashimoto’s thyroiditis.

**Table 1:** Novel loci identified in multi-ancestry GWAS meta-analysis of Hashimoto’s thyroiditis

Variant	Position	Annotated gene	Nearest gene	EAF	OR	*P* value
rs5912820-A	Xq21.1	*GPR174*	*GPR174*	0.85	1.09 (1.07–1.11)	3.45 × 10^−14^
rs2008260-A	14q32.2	*BCL11B*	*LINC01550*	0.20	1.07 (1.05–1.09)	2.43 × 10^−11^
rs75204333-T	16q22.1	*C16orf86*	*RIPOR1*	0.96	0.88 (0.85–0.92)	6.66 × 10^−11^
rs13134084-A	4q22.1	*AFF1*	*AFF1*	0.82	0.94 (0.92–0.96)	2.66 × 10^−10^
rs779542784-Del	16q12.1	*ADCY7*	*BRD7*	0.46	1.05 (1.04–1.07)	5.93 × 10^−10^
rs212411-A	6q25.3	*TAGAP*	*TAGAP*	0.55	1.05 (1.03–1.07)	6.50 × 10^−10^
rs13033698-A	2q36.2	*DOCK10*	*DOCK10*	0.65	1.05 (1.03–1.07)	1.10 × 10^−9^
rs6483293-T	11q21	*PANX1*	*PANX1*	0.61	1.05 (1.03–1.06)	1.73 × 10^−9^
rs7172006-A	15q25.3	*AKAP13*	*AKAP13*	0.34	1.05 (1.03–1.07)	3.47 × 10^−9^
rs11452540-Del	15q22.2	*CA12*	*LOC102723344*	0.70	0.95 (0.93–0.97)	5.53 × 10^−9^
rs35605052-Del	3p21.31	*FYCO1*	*LZTFL1*	0.18	1.06 (1.04–1.08)	7.03 × 10^−9^
rs4677734-T	3q28	*CCDC50*	*CCDC50*	0.40	1.05 (1.04–1.07)	1.12 × 10^−8^
rs221796-C	7q22.1	*GIGYF1*	*GIGYF1*	0.11	0.93 (0.91–0.95)	1.40 × 10^−8^
rs59384702-T	2q37.3	*SEPTIN2*	*HDLBP*	0.27	0.95 (0.94–0.97)	1.54 × 10^−8^
rs3946273-T	3q25.33	*SMC4*	*ARL14*	0.35	0.96 (0.94–0.97)	1.93 × 10^−8^
rs7516441-A	1p32.1	*FGGY*	*FGGY*	0.12	1.07 (1.05–1.10)	2.68 × 10^−8^
rs12223670-A	11q14.2	*HIKESHI*	*EED*	0.10	1.07 (1.04–1.09)	3.31 × 10^−8^
rs12118086-A	1p21.2	*S1PR1*	*S1PR1*	0.18	0.95 (0.93–0.97)	3.67 × 10^−8^
rs1926193-T	10q23.31	*FAS*	*FAS*	0.45	1.04 (1.03–1.06)	4.70 × 10^−8^

Variant, associated variant and effect allele; EAF, effect allele frequency; OR, odds ratio. All *P* values were obtained from two-sided association tests (*z*-statistics). The genome-wide significance level after multiple test corrections is *P* < 5 × 10^−8^.

Variants were mapped to the nearest gene using a UCSC Genome Browser data export (dbSNP v147) and additionally annotated by the Open Targets variant-to-gene (V2G) pipeline (v22.10). Among the novel associations shown in Table 1, we identified variants associated with genes involved in the immune response, including *TAGAP* and *DOCK10* implicated in T cell activation and differentiation, and other novel associations in major components of immune cell activation or cytokine production, including *PANX1*, *GPR174* and *BCL11B*^24,25^.

We also replicated associations mapped to genes like *NKX2-1*, which encodes a thyroid transcription factor regulating transcription of genes involved in thyroid hormone synthesis, including the thyroid stimulating hormone receptor (*TSHR*), thyroid peroxidase (*TPO*), thyroglobulin (*TG*) and the solute carrier family 26 member 4 (*SLC26A4*) encoding pendrin^26–29^. Other signals implicate genes known to be involved in thyroid hormone synthesis and signaling (*TPO*, *TG*, *PDE8B*) and represent well-established immune pathway associations (*LTA4H*), including cytokine-mediated signaling (*FLT3, IFITM3, IL2RA, IRF4, IRF5, JAK1, PTPN2, STAT4, TYK2*), T cell activation (*CD247, CD28, CTLA4, TNFRSF14, TNFRSF1B*) and major histocompatibility complex (MHC) genes (*HLA-A, HLA-B, HLA-C, HLA-DQA1, HLA-DQA2, HLA-DQB1, HLA-DPA1, HLA-DPB1, HLA-DRB5*)^2,16–19,22,23,30–32^.

### Fine-mapping results and ancestry-related heterogeneity

**Extended Data Fig. 1:**
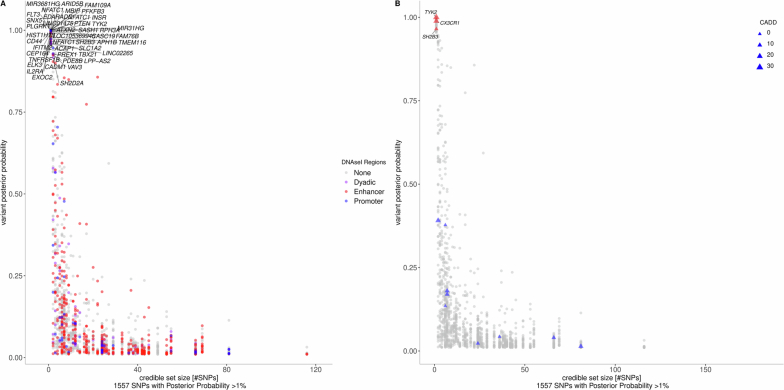
Fine-mapping and functional annotation of potentially causal variants. **a**,**b**, Overview of 1,557 variants with a posterior probability of > 1% of being a causal variant in each locus. The *x*-axis indicates the 95% credible set size and the *y*-axis the variants’ posterior inclusion probability. In **a**, variants are color-coded with respect to location in regulatory regions. In **b**, missense variants are marked by triangles, with size proportional to the SNP CADD score. The labels show the closest gene and are restricted to variants mapping to variants with high individual posterior probability (> 0.9) of driving the association signal.

We performed fine-mapping to distinguish between candidate causal variants and other variants in LD at associated loci using SuSiEx^33^ (Methods) by estimating credible sets that include the causal variant with a posterior inclusion probability of >95%. The number of variants in the credible sets ranged from 1 to 116 (Supplementary Table 4). We identified 93 variants with posterior inclusion probability of >0.80 in 59 independent loci (Supplementary Table 4). Most potential causal variants (posterior inclusion probability of >0.9) were located in regulatory regions (*n* = 44), including *ATXN2*, *PDE8B* and *VAV3*, whereas only three (*TYK2*, *CX3CR1* and *SH2B3*) were annotated as missense variants (Extended Data Fig. 1).

Using Meta-Regression of Multi-AncEstry Genetic Association (MR-MEGA), we examined the heterogeneity from our index variants to determine which of these are probably influenced by ancestral heterogeneity instead of other effects^34^. Six of our index variants (rs6679677, rs3130552, rs9272426, rs9273007, rs3892710 and rs9277799) showed significant heterogeneity owing to ancestry. All variants except rs6679677 (which is near *DCLRE1B*) are located within the MHC region known to vary widely across populations (Supplementary Table 3)^35^.

### European ancestry reference populations GWAS

**Extended Data Fig. 2:**
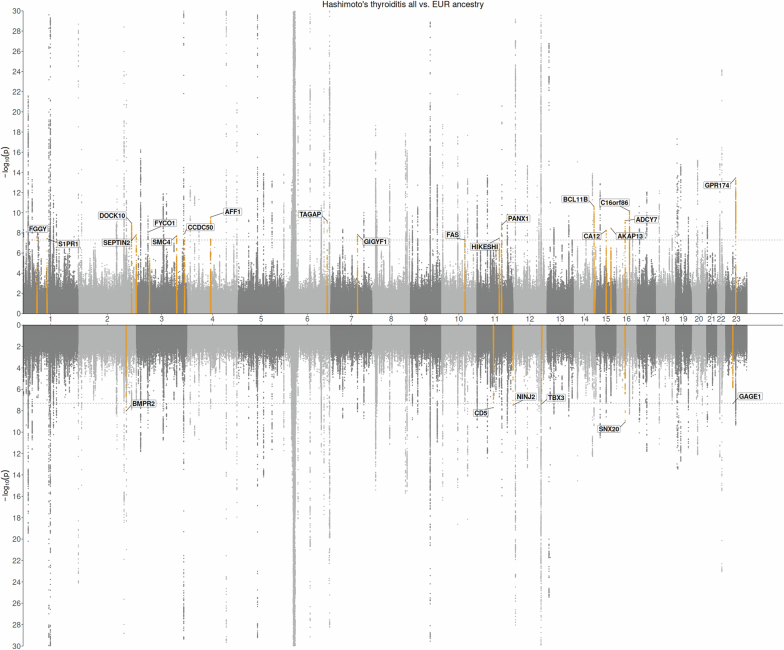
Multi-ancestry versus European-ancestry results. Mirrored Manhattan plots depict the association results for the multi-ancestry (top) vs. European-ancestry (bottom) meta-analysis. Chromosome numbers are depicted on the *x*-axis, with −log_10_(*P*-value) on the *y*-axis (cropped at 30). The grey line indicates genome-wide significance (*P* = 5 × 10^−8^). All *P*-values were obtained from two-sided association tests (*z*-statistics), where correction for multiple testing is indicated by the level of genome-wide significance. Stratum-specific novel loci are colored in orange and labeled by the annotated gene name.

We also performed a GWAS restricted to samples similar to European reference populations (*n* = 42,490 cases; 773,420 controls) in which we identified six loci that were not captured in the multi-ancestry GWAS at *CD5*, *TBX3*, *NINJ2*, *SNX20*, *BMPR2* and *GAGE1*, suggesting risk loci specific to the European reference populations (Supplementary Table 5 and Extended Data Fig. 2). In summary, we identified variants like rs142997491 (*SNX20*) and rs148481877 (*CD5*) with ancestry-specific effect sizes or that were monomorphic in non-European reference populations and the East Asian ancestry population GWAS.

To assess whether there might be potential environmental effects that could lead to the ancestry-specific results, we checked for pleiotropy effects in the GWAS Catalog (Supplementary Table 6). Results included pleiotropic effects of the index variants or their proxies (*r*^2^ ≥ 0.8) with smoking behavior, socioeconomic traits and educational attainment (rs1477031: *BMPR2*), but also with type 1 diabetes (rs148481877: *CD5*) and other autoimmune diseases (rs5906843: *GAGE1*).

### Sex-specific susceptibility loci

**Fig. 3:**
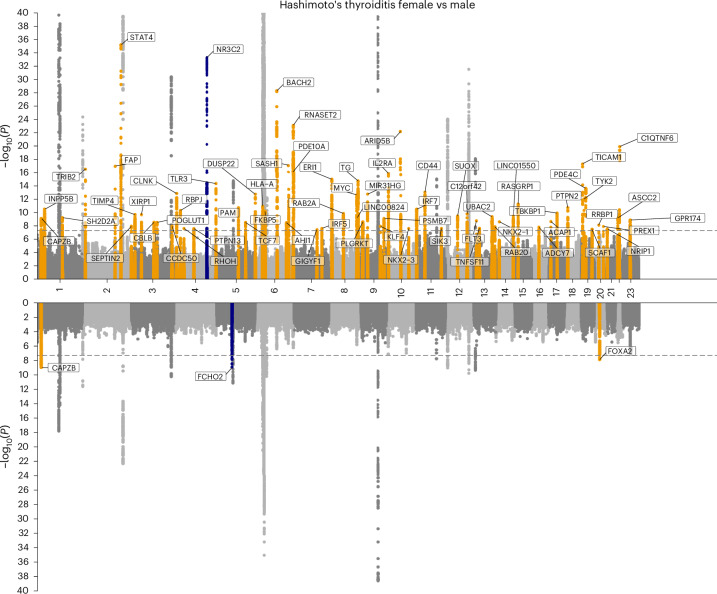
Sex-stratified genome-wide association results for Hashimoto’s thyroiditis. Mirrored Manhattan plots depict the association results for the female (top) and male (bottom) meta-analysis. Chromosome numbers are depicted on the *x* axis, with −log_10_(*P* value) on the *y* axis (cropped at 40). The gray line indicates genome-wide significance (*P* = 5 × 10^−8^). All *P* values were obtained from two-sided association tests (*z*-statistics), where correction for multiple testing is indicated by the level of genome-wide significance. Stratum-specific loci are colored in orange and labeled by the annotated gene name. The blue loci highlight the significant variants from the sex-interaction analysis. Of note, the *CAPZB* locus on chromosome 1 has independent sex-specific index variants (Supplementary Fig. 2).

**Extended Data Fig. 3:**
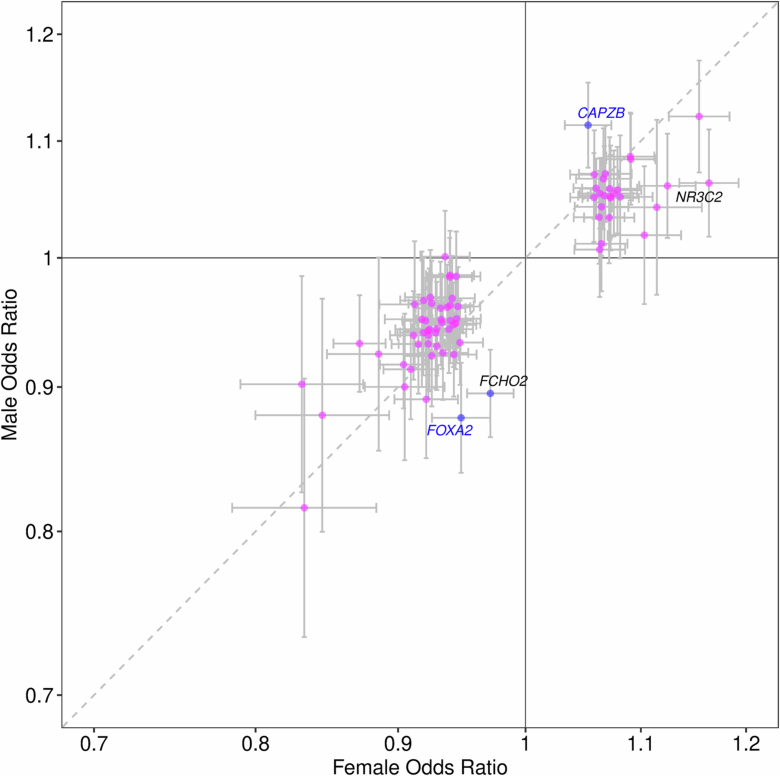
Effect sizes of the sex-specific GWAS results. Comparison of the GWAS association effect sizes (odds ratios) between *n* = 594,184 females (*x*-axis) and *n* = 498,123 males (*y*-axis) of the significant sex-specific GWAS associations (*P* < 5 × 10^−8^). Purple dots indicate female, and blue dots and labels male-specific results. The variants with black labels indicate the significant sex-interactions (*P* < 3.96 × 10^−4^). All *P*-values were obtained from two-sided association tests (*z*-statistics). Error bars show 95% confidence intervals of the odds ratios. The dashed line represents the diagonal.

**Extended Data Fig. 4:**
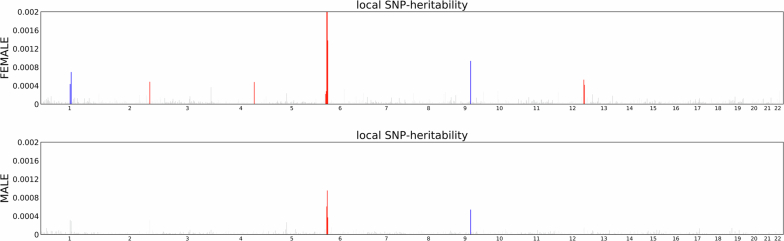
Local SNP-heritability of females and males. The local heritability based on the GWAS results from females (upper panel) and males (lower panel) estimates using the HESS software (https://huwenboshi.github.io/hess-0.5/). Loci that have significant local SNP-heritability are colored in red and blue. In addition to males, females have significant local heritability estimates for Hashimoto’s thyroiditis on chromosomes 1, 2, 4, and 12. Source data

We additionally performed sex-specific GWAS meta-analyses of Hashimoto’s thyroiditis using multi-ancestry GWAS summary statistics from 37,850 female cases and 556,334 female controls and 10,799 male cases and 487,324 male controls. In total, 108 independent variants in 84 loci in females and 20 independent variants in 18 loci in males were significantly associated (Fig. 3 and Supplementary Tables 7 and 8). There were three significant associations (rs12089835: *CAPZB*; rs10068965: *FCHO2*; and rs11480708: *FOXA2*) in males that were not significant in females, even though there was higher power in the female-specific GWAS (Fig. 3 and Supplementary Fig. 2). Using previously described methods^36^ by comparing the effect size and variance in effect size between GWAS, we uncovered two distinct variants exhibiting significant sexual dimorphism in their associations with Hashimoto’s thyroiditis at *P* < 3.96 × 10^−4^ (0.05 / 126 independent tests) (Supplementary Table 9). The first single nucleotide polymorphism (SNP), rs10068965 located on chromosome 5 at the *FCHO2* locus, was associated exclusively in males (*P*_male_ = 1.18 × 10^−9^) while remaining non-significant in females (*P*_female_ = 0.003) or the combined-sex multi-ancestry GWAS results (*P* = 1.09 × 10^−7^). Conversely, the second SNP, rs7695810 on chromosome 4 near *NR3C2* encoding the mineralocorticoid receptor, had a significant association in females (*P*_female_ = 4.99 × 10^−34^), with no significant signal observed in males alone (*P*_male_ = 6.18 × 10^−3^). The effect sizes of the significant sex-specific loci are contrasted in Extended Data Fig. 3. The heritability on the liability scale related to all GWAS variants in the overall sample was *h*^2^ = 10.5% (Hashimoto’s thyroiditis prevalence, 4%) and comparable to a previous GWAS using the same LDSC approach^17^. However, this value was substantially higher in females (*h*^2^ = 11.8%; Hashimoto’s thyroiditis prevalence, 6%) than in males (*h*^2^ = 8.8%; Hashimoto’s thyroiditis prevalence, 2%). This difference was also reflected by more chromosomal regions in the female GWAS that showed significant local SNP heritability estimated using HESS^37^ (Extended Data Fig. 4). These results highlight a higher genetic predisposition for Hashimoto’s thyroiditis in females that could partly explain the sex-specific differences in disease prevalence.

### In silico characterization of Hashimoto’s thyroiditis-associated variants

All subsequent follow-up analyses were based on the multi-ancestry sex-combined results to maximize statistical power for exploring the functional and clinical consequences of the identified Hashimoto’s thyroiditis associations.

### Gene pathway and tissue expression enrichment analyses

**Extended Data Fig. 5:**
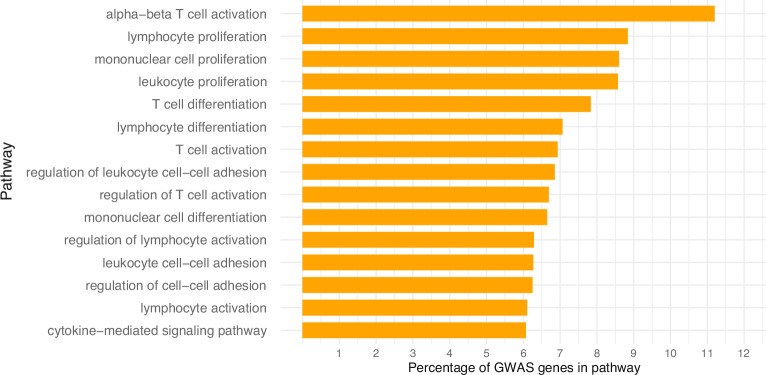
Top 15 pathways enrichment results. The top 15 associated enrichment pathways for the significant multi-ancestry GWAS findings, excluding the MHC region, are shown (*y*-axis). As all top pathways achieved the same significant *P*-value by a one-sided permutation test (Methods), the results were ordered by their percentage of GWAS genes matched to the respective pathway (*x*-axis). All pathways displayed belong to the biological processed of the gene ontology.

We used Gene Ontology and the KEGG database to test for enrichment of pathways with genes assigned to the 155 significantly associated variants. The 495 significantly enriched pathways included activation and regulation of leukocytes and their subtypes, interferon signaling and response and pathways related to autoimmune diseases and cancer. These results were similar after excluding the MHC region with its human leukocyte antigen (HLA)-related genes (Supplementary Table 10 and Extended Data Fig. 5). With a few exceptions, like the endocrine system and gland development, all of the enriched pathways are involved in immune and inflammation-related processes.

**Fig. 4:**
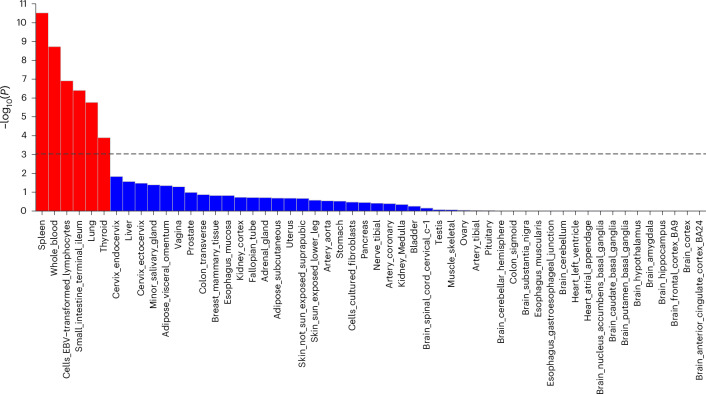
MAGMA tissue enrichment resulting in 54 GTEx tissue types. Nominal −log_10_(*P* values) were based on a one-sided positive *t*-test and are shown on the *y* axis, the respective tested tissues on the *x* axis and the dotted line shows the Bonferroni-corrected significance threshold (*P* = 9.4 × 10^−4^). Red bars show significant tissue enrichment results; blue bars show non-significant results.

We performed multiple tissue and cell type enrichment analyses to test whether the genes associated with Hashimoto’s thyroiditis variants are significantly enriched in their expression in specific tissues. Multi-marker Analysis of GenoMic Annotation (MAGMA) within Functional Mapping and Annotation of Genome-Wide Association Studies (FUMA) assigns all Hashimoto’s thyroiditis variants to the nearest gene and tests whether these genes are enriched in expression in 54 GTEx tissues^38,39^. We identified significantly enriched expression with a Bonferroni-adjusted *P* < 9.3 × 10^−4^ (0.05 / 54 independent tests) in six tissues: spleen, whole blood (Epstein–Barr virus-transformed) lymphocytes, terminal ileum of the small intestine, lung and thyroid (Fig. 4 and Supplementary Table 11). Notably, the spleen and whole blood represent crucial components of the immune system, whereas the small intestine and lung enrichment probably stems from their immune-related functions through environmental exposures.

**Extended Data Fig. 6:**
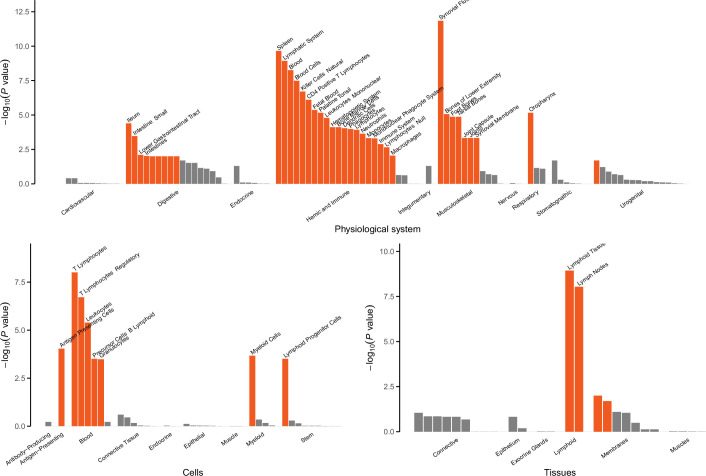
DEPICT tissue, cell, and physiological system enrichment analyses. Tissue-enrichment analyses using DEPICT are shown for analyses based on physiological systems, cell types as well as tissues (*x*-axis) with –log_10_(*P*-values) for the enrichment on the *y*-axis. *P*-values were obtained from two-sided *z*-tests (Methods). Significant enrichments after correcting for a false discovery rate are depicted in red.

We used Data-Driven Expression Prioritized Integration for Complex Traits (DEPICT) as a complementary analysis to MAGMA and identified a similar set of enriched tissues as the MAGMA results with more resolution. DEPICT tissue enrichment identified the digestive system, including the small intestine and specifically the ileum; the hematologic and immune system, including the spleen, blood, natural killer cells and CD4^+^ T cells; and the musculoskeletal system, including synovial fluid (Extended Data Fig. 6). DEPICT also identified enriched cell types: T cells, antigen-presenting cells, precursor B cell lymphoid and lymphoid progenitor cells, as well as granulocytes and myeloid cells. Taken together, enrichment was predominantly observed in tissues and cells involved in the immune system (Supplementary Table 12).

### Single-cell RNA-based analyses

As gene expression enrichment of the GWAS loci was observed in both immune-related tissues as well as in the thyroid, we used LDSC regression for Specifically Expressed Genes (LDSC-SEG) based on gene expression data to determine whether genes highly expressed in specific cell types were enriched in SNP-based heritability^40,41^. Applying this method to the eight broad thyroid cell types from single-cell RNA sequencing (scRNA-seq) of healthy thyroid glands (*n*_thyroid_ = 7), the T cell and natural killer cell group was significantly (0.05 / 8 cell types = 6.25 × 10^−3^) enriched for Hashimoto’s thyroiditis-associated genetic variants (*P* = 5.01 × 10^−6^) (Supplementary Table 13).

**Extended Data Fig. 7:**
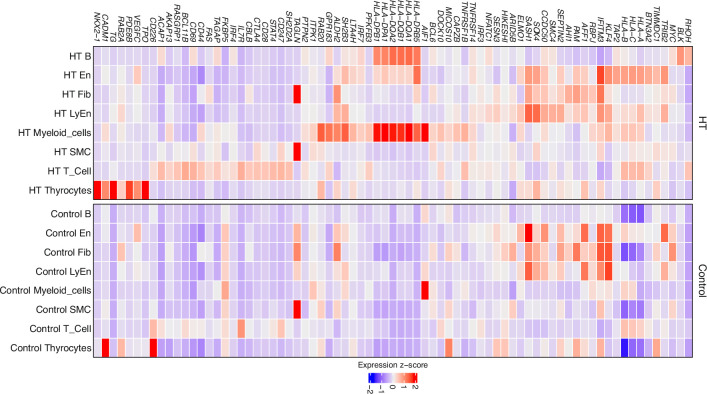
Heatmap of significantly differentially expressed genes between Hashimoto’s thyroiditis cases and controls. Heatmap of the 75 significantly differentially expressed genes (*x*-axis) between Hashimoto’s thyroiditis (HT) cases and controls among the tissues (*y*-axis) in the scRNA-seq dataset. Source data

Following this step, we examined the further categorized 11 immune cell types within the thyroid and identified activated and naive CD4^+^ T cells to be significantly (*P* < 0.05 / 11 cell types = 4.5 × 10^−3^) enriched (Supplementary Table 13). Additionally, we applied this method to circulating immune cells from the Database of Immune Cell eQTLs, expression and epigenomics (DICE) and identified significant enrichment of variants (*P* < 0.05 / 15 cell types = 3.3 × 10^−3^) in activated CD4^+^ and CD8^+^ T cells and T follicular helper cells (*P* = 5.8 × 10^−3^, 3.3 × 10^−3^ and 4.4 × 10^−3^, respectively) (Supplementary Table 14). To assess whether and how these results translate to the thyroid tissue of patients with Hashimoto’s thyroiditis, we ran LDSC-SEG on an additional scRNA-seq dataset combining two patients with Hashimoto’s thyroiditis and one control. We found a significant enrichment of Hashimoto’s thyroiditis-associated variants in T cells from the thyroid tissue of the patient with Hashimoto’s thyroiditis after Bonferroni correction (*P* < 0.05 / 16 cell types = 3.3 × 10^−3^) and a weaker but nominally significant effect for this cell type in the control sample (Table 2). Of note, 75 out of our 155 Open Targets annotated GWAS genes (Supplementary Table 3) were differentially expressed in this scRNA-seq dataset (Extended Data Fig. 7).

**Table 2:** Heritability enrichment of thyroid-specific cell populations from Hashimoto’s thyroiditis cases and controls

Cell type	Coefficient	Coefficient SE	Coefficient *P*
**T cells (Hashimoto’s thyroiditis)**	**5.84** **×** **10**^**−9**^	**1.39** **×** **10**^**−9**^	**1.33** **×** **10**^**−5**^
*T cells (control)*	*1.80* *×* *10*^*−9*^	*9.95* *×* *10*^*−10*^	*0.04*
B cells (Hashimoto’s thyroiditis)	1.29 × 10^−9^	9.55 × 10^−10^	0.09
B cells (control)	−9.94 × 10^−10^	6.90 × 10^−10^	0.93
Endothelial cells (Hashimoto’s thyroiditis)	−1.19 × 10^−9^	8.58 × 10^−10^	0.92
Endothelial cells (control)	−5.64 × 10^−10^	7.09 × 10^−10^	0.79
Fibroblasts (Hashimoto’s thyroiditis)	−9.15 × 10^−10^	8.12 × 10^−10^	0.87
Fibroblasts (control)	1.48 × 10^−10^	8.27 × 10^−10^	0.43
LyEn (Hashimoto’s thyroiditis)	−1.86 × 10^−10^	9.64 × 10^−10^	0.58
LyEn (control)	−9.34 × 10^−10^	7.93 × 10^−10^	0.88
Myeloid cells (Hashimoto’s thyroiditis)	2.50 × 10^−10^	8.97 ×10^−10^	0.39
Myeloid cells (control)	−1.28 × 10^−9^	8.95 × 10^−10^	0.92
SMC (Hashimoto’s thyroiditis)	1.29 × 10^−10^	9.93 × 10^−10^	0.45
SMC (control)	−6.47 × 10^−10^	6.11 × 10^−10^	0.86
Thyrocytes (control)	−1.18 × 10^−9^	6.75 × 10^−10^	0.96
Thyrocytes (Hashimoto’s thyroiditis)	1.68 × 10^−9^	1.17 × 10^−9^	0.08

Cell type enrichment that passed the Bonferroni *P* value threshold (*P* < 0.0031) depicted in bold; nominally significant (0.0031 < *P* < 0.05) cell type enrichments in italic. The coefficient column represents the increase in per-SNP heritability associated with the given cell type conditional on all cell types and other annotations in the baseline model (including genic regions, enhancer regions and conserved regions). The coefficient *P* value shows whether the coefficient is significantly positive and was used to assess tissue enrichment. *P* values are one-sided and were obtained from a correlation test, with uncertainty subsequently estimated by block jackknife (*z*-statistics).

### Colocalization

**Extended Data Fig. 8:**
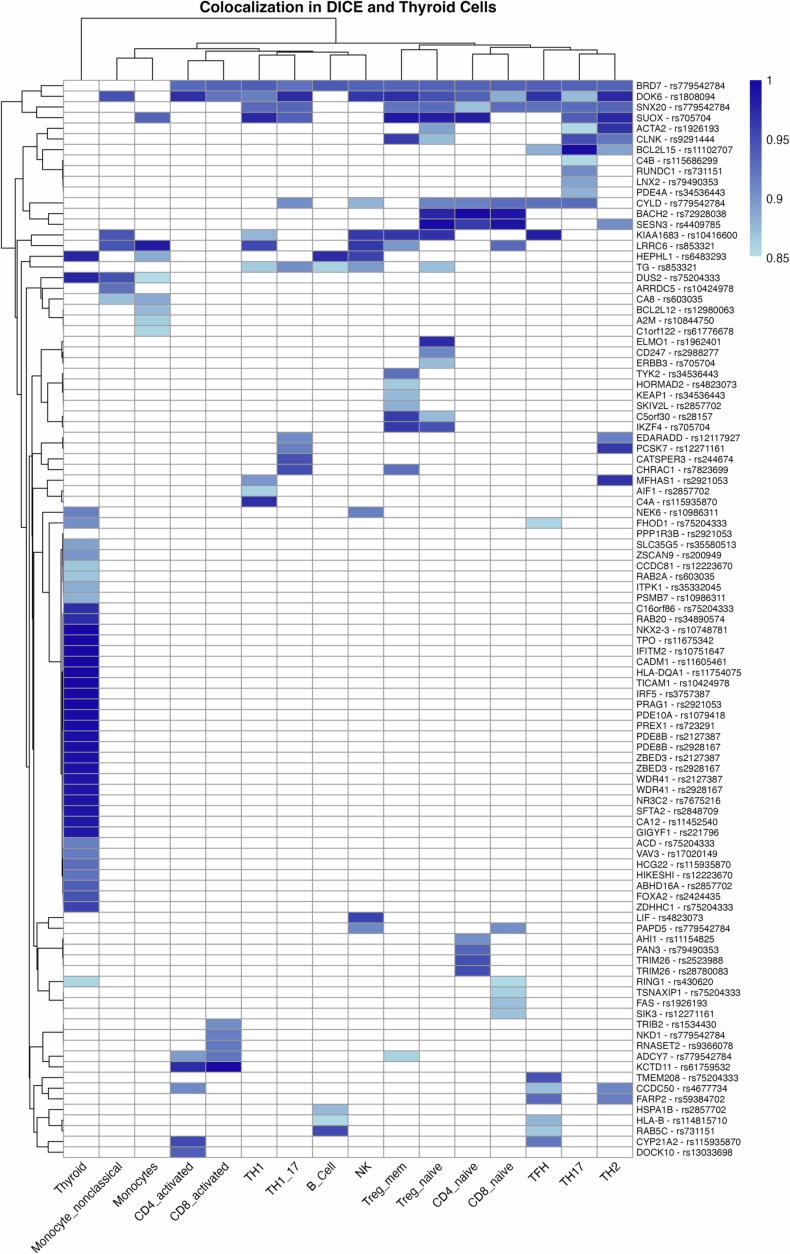
Colocalization of Hashimoto’s thyroiditis association signals for thyroid tissue and immune cells. The heatmap illustrates colocalized genes across thyroid tissue and various immune cells from DICE and GTEx datasets. The tissues or cell types are labeled on the bottom *x*-axis, and the gene names and RSID associated with the eQTL of interest are labeled on the *y*-axis. Colocalization of genes between tissues with a posterior probability > 0.85 are depicted in blue varying from light to dark blue indicating higher posterior probability. Colocalizations with a posterior probability < 0.85 are left blank.

Next, we assessed whether significant genetic variants affect the risk of Hashimoto’s thyroiditis through differential gene expression. To do so, we conducted colocalization analyses with known expression quantitative loci (eQTLs) from publicly available databases focusing on tissues and cell types identified through our enrichment analyses. We included thyroid tissue and whole blood (representing circulating immune cells) from the GTEx Project database^42^. Furthermore, we used 15 immune cell types obtained from DICE^43^. In total, we found 40 significant colocalizations (posterior probability of >0.85) in thyroid tissue and 21 in whole blood from GTEx v8, and 161 colocalizations in the immune cells from the DICE dataset (Extended Data Fig. 8 and Supplementary Table 15). These colocalizations showed a distinct pattern between thyroid and immune cells. The colocalizations specific to thyroid tissue encompassed several genes integral to thyroid hormone production and regulation, including *TPO, PDE10A* and *PDE8B*. Interestingly, eQTLs in the *TG* gene were found in the immune cells only. Furthermore, multiple colocalizations in the thyroid were identified in immune regulatory genes, including *VAV3, IRF5, HLA-DQA1, CADM1* and *AIF1*, highlighting the interplay between the immune system and the thyroid. Two genes colocalized in almost all (13 out of the 15) immune cell types: bromodomain containing 7 (*BRD7*), which is involved in tumor growth prevention, and docking protein 6 (*DOK6*), which has a role in the RET signaling cascade.

### Genetic correlations

**Fig. 5:**
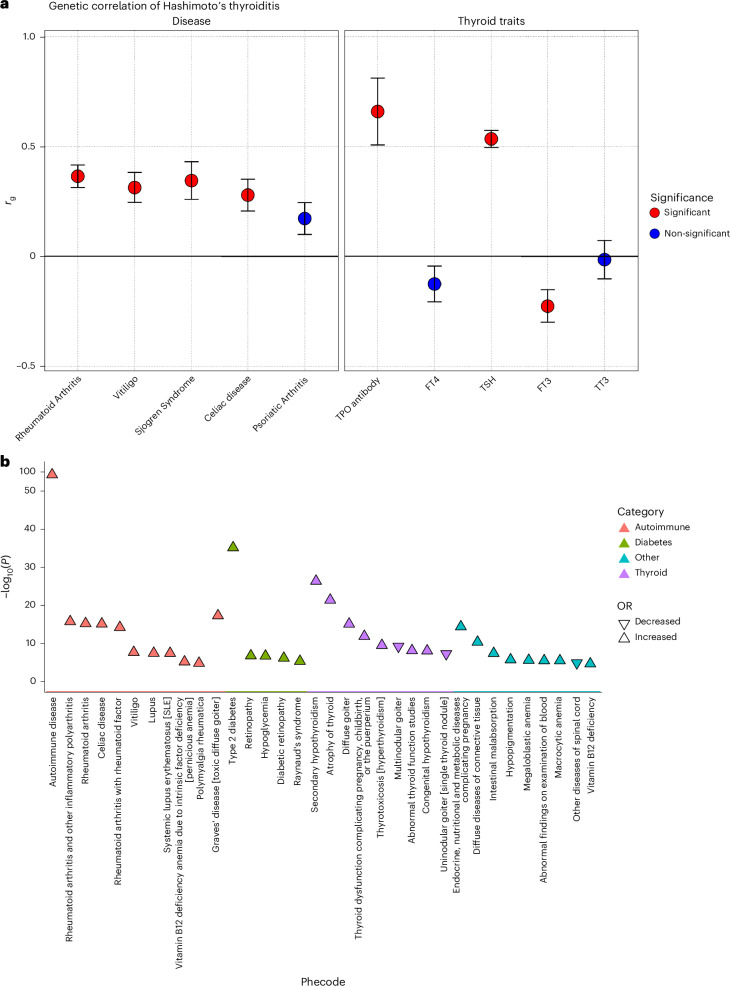
Genetic similarity between Hashimoto’s thyroiditis and other autoimmune diseases and thyroid-function-related traits. **a**, The plot illustrates the genetic correlation of Hashimoto’s thyroiditis with the top five comorbid autoimmune diseases and thyroid traits, including TPO antibody status, TSH, free T4 (FT4), total T3 (TT3) and free T3 (FT3). The *x* axis represents the tested phenotypes for genetic correlation, while the *y* axis indicates the genetic correlation estimate. Dots denote the *r*_g_ value, and error bars represent the corresponding s.e.m. The *r*_g_ is based on an average of 997,655 genetic variants across the traits (Supplementary Table 16). The color of the dots indicates significance, with red indicating Bonferroni-corrected significant correlations obtained by two-sided *z*-tests (*P* < 0.005), and blue indicating non-significant correlations. **b**, The plot displays the Bonferroni-corrected significant associations (–log_10_(0.00003162) = 4.5) from the phenome-wide association study results from the *All of Us* database obtained by two-sided association tests (*z*-statistics). Categories are labeled on the *x* axis, and –log_10_(*P*) is depicted on the *y* axis. Each triangle on the plot corresponds to the associated phenotype for the respective category and points up or down, corresponding to the direction of the effect.

Genetic correlations (*r*_g_) between Hashimoto’s thyroiditis and the top five most common comorbid autoimmune diseases (rheumatoid arthritis, Sjögren syndrome, celiac disease, vitiligo and psoriatic arthritis), TPOAb status and thyroid hormone levels (TSH, free T4, free T3 and total T3) were calculated using recently published GWAS summary statistics for each trait^19,44–50^. Genetic correlations were considered significant after Bonferroni adjustment for multiple testing (*P* < 0.05 / 10 traits = 0.005). Significant genetic correlations were observed between Hashimoto’s thyroiditis and autoimmune diseases: rheumatoid arthritis (*r*_g_ = 0.365, *P* = 1.11 × 10^−12^), vitiligo (*r*_g_ = 0.313, *P* = 5.33 × 10^−6^), Sjögren syndrome (*r*_g_ = 0.345, *P* = 5.68 × 10^−5^), celiac disease (*r*_g_ = 0.278, *P* = 1.00 × 10^−4^) and thyroid hormone levels: TSH (*r*_g_ = 0.535, *P* = 5.07 × 10^−43^), free T3 (*r*_g_ = −0.226, *P* = 0.0024) and TPOAb status (*r*_g_ = 0.659, *P* = 1.39 × 10^−5^) (Fig. 5a and Supplementary Table 16). No significant genetic correlations after multiple-testing correction were observed with psoriatic arthritis, free T4 or total T3.

### Phenome-wide association study

We used data from the *All of Us* Research Program to perform an agnostic phenome-wide association study (PheWAS) using polygenic scores across 1,581 diverse phenotypes^51^. The initial PheWAS was based on Hashimoto’s thyroiditis-associated results, excluding the MHC region, and identified significant associations (*P* < 0.05 / 1,581 phenotypes = 3.16 × 10^−5^) with 25 different phenotypes, predominantly other autoimmune diseases (Supplementary Table 17). Among those are Graves’ disease, type 1 diabetes, celiac disease, systemic lupus erythematosus and rheumatoid arthritis. Additionally, various thyroid-related associations, such as secondary hypothyroidism, diffuse goiter, multinodular goiter, thyrotoxicosis (hyperthyroidism), atrophy of the thyroid, as well as malignant neoplasm of the skin, were observed. Extending the PheWAS by including the MHC region GWAS results revealed 34 associations in total, including additional ones with anemia, hypoglycemia and intestinal malabsorption, but without type 1 diabetes and melanoma (Fig. 5b and Supplementary Table 17). The genetic correlation and PheWAS results emphasize a common underlying genetic profile of Hashimoto’s thyroiditis and numerous other autoimmune diseases.

### Mendelian randomization

**Fig. 6:**
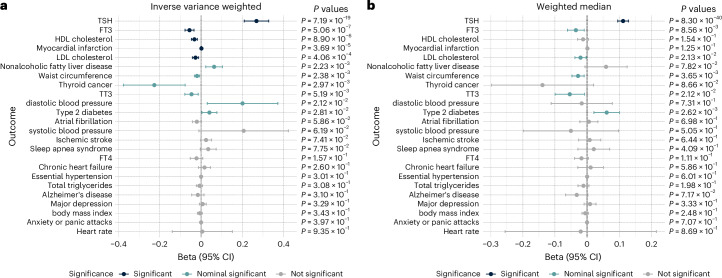
Causal effects of Hashimoto’s thyroiditis on thyroid-function-related traits and clinical outcomes using MR. **a**,**b**, Effects from MR analysis with genetic predisposition to Hashimoto’s thyroiditis as exposure. The dots on the *x* axis represent the causal effect estimate with its corresponding error bars indicating the 95% upper and lower confidence intervals (CI), respectively, and the *y* axis denotes the outcomes (clinical parameters or diseases). Dot colors denote significance levels: dark blue for outcomes below the Bonferroni-corrected significance threshold (*P* < 0.002) obtained by two-sided *z*-tests, light blue for nominal (uncorrected) significance (0.002 < *P* < 0.05) and gray for non-significant outcomes (*P* > 0.05). Results are shown for the inverse variance-weighted analysis (**a**) and weighted median analysis (**b**). The median number of SNPs included in the MR analyses was 137 (Supplementary Table 18).

We performed two-sample Mendelian randomization (MR) to test for causal relationships between genetic predisposition to Hashimoto’s thyroiditis (exposure) and 24 common, clinically related diseases and measurements (outcomes) (Methods)^52^. Datasets that were primarily composed of UK Biobank samples were not included, as those overlapped with the data used in this GWAS. Results that met a significance threshold of *P* < 2.08 × 10^−3^ (0.05 / 24 independent tests) were considered significant. Inverse variance-weighted MR analyses revealed significant effects (*P* < 2.08 × 10^−3^) on higher TSH levels and an increased risk of myocardial infarction, and lower levels of free T3, high-density lipoprotein (HDL) and low-density lipoprotein (LDL) cholesterol. Nominally significant effects (*P* < 0.05) were detected for Hashimoto’s thyroiditis for a lower risk of thyroid cancer, and for lower waist circumference and total T3 levels, but higher diastolic blood pressure, and an increased risk for type 2 diabetes and non-alcoholic fatty liver disease (Fig. 6 and Supplementary Table 18). However, some traits indicated a high degree of heterogeneity owing to pleiotropy, suggesting that these results may be inconclusive. As a sensitivity analysis, we applied the pleiotropy-robust but less powerful weighted median MR, which revealed consistent effect directions with the inverse variance-weighted approach. In addition, the sensitivity analysis results confirmed potential causal effects on TSH (*P* = 8.3 × 10^−40^), free T3 (*P* = 0.009) and LDL cholesterol (*P* = 0.021).

## Discussion

To our knowledge, this is the first Hashimoto’s thyroiditis GWAS meta-analysis containing mixed-ancestry populations, in contrast to previous GWAS studies in predominantly European ancestry populations^16,17^. Previous studies used a less precise Hashimoto’s thyroiditis case definition and mainly used publicly available data that were not specific for Hashimoto’s thyroiditis, including other causes of hypothyroidism^19,53,54^. One recent GWAS used Hashimoto’s thyroiditis and Graves’ disease diagnoses combined, even though they have been shown to have unique genetic profiles and present opposite levels of thyroid hormones (that is, hypothyroidism vs hyperthyroidism)^55^. By using a specific case definition limited to autoimmune hypothyroidism, we identified 155 independent variants associated with Hashimoto’s thyroiditis, including 45 novel signals. The majority of the associations map to genes involved in immune regulation, such as HLA, cytokine and cytokine receptor genes, which aligns with the well-established role of immune-mediated mechanisms in Hashimoto’s thyroiditis pathophysiology^18,19,53,54^. We have demonstrated that a more specific case definition potentiates the identification of unique genetic associations and molecular pathways for this most common form of hypothyroidism.

Our study revealed ancestry-specific genetic variants, including two alleles present in low frequency in European ancestry populations but absent in East Asian ancestry populations (rs142997491 in *SNX20*, rs148481877 in *CD5*). These results suggest that Hashimoto’s thyroiditis genetic risk is similar across ancestral backgrounds, with some associations specific to certain populations. This underscores the importance of more diverse genetic sampling in the future to better understand the similarities and differences in genetic risk between different ancestries. Additionally, in the sex-specific GWAS, three variants (rs12089835: *CAPZB*; rs10068965: *FCHO2*; and rs11480708: *FOXA2*) were significantly associated in males but not in females, even though we had higher power in females. Although rs12089835 and rs11480708 variants have been previously associated with Hashimoto’s thyroiditis, here we show that these associations are probably driven by males^17^. There were a total of 70 variants significantly associated in females that did not reach the significance threshold in males, but as we had almost three times the number of female cases as males, this finding might be explained by differences in statistical power. Therefore, we performed an interaction analysis comparing effect size and variance and found that two variants were significantly different, including a female-specific variant near *NR3C2* (rs7695810) and a novel male-specific variant (rs10068965) at *FCHO2. FCHO2* is involved in clathrin-mediated endocytosis and is ubiquitously present in the thyroid. Endocytosis is involved in various aspects of immune function, including antigen presentation, cytokine signaling, receptor trafficking and the uptake and transport of thyroglobulin in thyroid cells, making this association a good candidate for follow-up studies^56^. Variation (rs7695810) in *NR3C2* has previously been associated with Hashimoto’s thyroiditis, but here we show that this is more pronounced in females. Additionally, this SNP has been described as a protein-QTL of thyroid stimulating hormone subunit beta (*TSHB*)^57^. Overall, the identification of ancestry-specific and sex-specific associations illustrates the importance of considering population diversity in genetic studies of hypothyroidism.

When testing for SNP enrichment within the target tissue of interest, the thyroid, we found enrichment in the resident CD4^+^ T cells of the thyroid. scRNA-seq analyses showed that this enrichment in immune cells was more pronounced in Hashimoto’s thyroiditis thyroids compared to healthy thyroids. CD4^+^ T cells represent a large group, including T_H_1, T_H_2, T_H_17, and regulatory T cells. In Hashimoto’s thyroiditis, following initial thyroid damage, CD4 T cells recognize thyroid antigens, such as thyroglobulin and TPOAb, as foreign and mount an immune response against them^7^. These activated CD4^+^ T cells release pro-inflammatory cytokines, ultimately promoting antibody production by B cells. The enrichment analysis using circulating immune cells from DICE additionally identified activated CD8^+^ T cells and T follicular helper cells, while also confirming the CD4^+^ T cell association. T follicular helper cells can contribute to the pathogenesis of Hashimoto’s thyroiditis through various mechanisms, such as in the formation and maintenance of thyroid germinal centers, which are specialized microenvironments where B cells undergo rapid proliferation, somatic hypermutation of antibody genes and stringent selection for high-affinity antigen binding^7^. T follicular helper cells also produce cytokines, including interleukin-21, which is critical for B cell differentiation and antibody production.

We also detected associations between Hashimoto’s thyroiditis variants and several eQTLs using colocalization analysis, including several genes integral to thyroid hormone production and regulation and many immunoregulatory genes expressed in thyroid cells. These findings suggest that many of our variants could be causal and highlight the interplay between the thyroid and immune cells. These serve as candidate variants and genes for functional follow-up in vitro analyses. Additionally, the genes associated with the eQTLs were analyzed to identify potential drug targets (see Supplementary Note 2), as no treatment currently exists for the underlying immune response in Hashimoto’s thyroiditis.

Genetic correlation analyses revealed shared genetic architecture between Hashimoto’s thyroiditis and common coinciding autoimmune disorders such as vitiligo, celiac disease, Sjögren syndrome and rheumatoid arthritis, as well as the thyroid-function-related traits TSH, free T3 and TPOAb status. PheWAS analyses replicated associations with the same autoimmune diseases and additionally identified systemic lupus erythematosus, pernicious anemia and Graves’ disease. Additionally, numerous diabetes-related associations emerged, including type 1 and type 2 diabetes, hypoglycemia and diabetic retinopathy. These results together confirm the presence of a shared immune mechanism between many different autoimmune diseases.

In addition to pleiotropic associations, we used two-sample MR to test whether genetic predisposition to Hashimoto’s thyroiditis has a causal effect on related clinical outcomes. The results show that Hashimoto’s thyroiditis has a potential causal effect on the components relevant for the development of metabolic syndrome. Interestingly, the inverse effect on LDL cholesterol is opposite to what would be expected. Given the substantial heterogeneity that was observed among the instruments, these results may be driven predominantly by autoimmune-related genetic variants. Our MR analyses also suggest that Hashimoto’s thyroiditis may have a significant protective effect on the development of thyroid cancer. Whether Hashimoto’s thyroiditis provides a protective or risk effect on thyroid cancer has long been debated^58,59^. Recently, MR analyses using a large TSH GWAS suggested that higher TSH levels are associated with a lower risk of thyroid cancer^49^. We reason that underlying genetics has an important role in the risk and/or protection against thyroid cancer, as shown in the polygenic risk score analyses performed on TSH levels and thyroid cancer^49^. This information could also be further leveraged in future personalized medicine to better assess an individual’s risk for thyroid cancer. Small sample sizes with different genetic backgrounds may also help explain why different observational studies obtained conflicting results assessing the relationship between Hashimoto’s thyroiditis and thyroid cancer^58,59^.

There are limitations to this study, including limited sample sizes of non-European ancestry groups (Supplementary Table 1). To address this issue, it is crucial to bolster genetic collections in underrepresented populations and expand diversity in imputation reference panels. Additionally, our results were limited to the analysis of common and low-frequency variants. Future studies using whole-genome sequences could be useful in identifying rare variants contributing to Hashimoto’s thyroiditis.

In conclusion, this study advances our understanding of the genetic basis of Hashimoto’s thyroiditis, uncovering novel associations in immune-related pathways, tissues and cells in the blood and within the thyroid, while also revealing population-specific genetic associations and sex-specific effects that provide valuable insights into Hashimoto’s thyroiditis pathogenesis. Furthermore, our analyses highlight the shared genetic basis of Hashimoto’s thyroiditis with other common autoimmune diseases, as well as the causal associations with various clinical outcomes.

## Methods

Our research complies with all relevant ethical regulations, all participants in this project gave informed consent and individual studies were approved by the cohort-specific ethics committees; that is, the *All of Us* Institutional Review Board; research ethics committees at the Institute of Medical Science, the University of Tokyo, the RIKEN Yokohama Institute; Ethics Committees of the University of Split, School of Medicine and the University Hospital Split; MyCode Governing Board; Estonian Committee on Bioethics and Human Research; NHS Health Research Authority: East of Scotland Research Ethics Service; The Central Medical Ethics Committee of Latvia; Mid-Norway Regional Committee for Medical and Health Research Ethics; University of Michigan Institutional Review Board; NHS Health Research Authority: North West–Haydock Research Ethics Committee.

### Trait definition

Autoimmune hypothyroid cases and controls were identified using the *International Classification of Diseases* 9^th^ (ICD9) and 10^th^ (ICD10) version for each cohort (slightly adapted for the HUNT study). Participants with autoimmune thyroiditis ICD9/10 codes 245.2/E06.3 and hypothyroidism, unspecified 244.9/E03.9 were selected as cases in our GWAS (Supplementary Table 2). Only studies having at least 100 cases were considered in the GWAS. Known potential causes of hypothyroidism were excluded from being cases or controls, such as other forms of hypothyroidism, thyroiditis, hyperthyroidism, thyroid cancer (medical history or current), post-partum thyroiditis, thyroid interfering medication (lithium or amiodarone), medical history of thyroid surgery or medical history of radioactive iodine treatment (Supplementary Table 2).

### GWAS in individual studies

In each study, genotyping using genome-wide arrays was conducted, followed by imputation of the genome-wide data to high-density imputation reference panels (Supplementary Table 1). Logistic regression was performed by adjusting for sex, age and age^2^. Additional covariates included genetic principal components to address population stratification within individual cohorts, relatedness, study site, residence, field center or laboratory batch, where applicable (Supplementary Table 1). Details on genotyping and imputation are provided in Supplementary Table 1. The meta-analyses included data from ten independent cohorts with 48,694 cases and 1,044,134 controls (Supplementary Table 1).

### Meta-analyses of individual cohort results

The files containing the results from the studies included in the meta-analyses were subjected to a thorough quality control process. This involved checking the format of the files, removing duplicate variants and identifying associations with missing or invalid values. The alleles of the SNPs and INDELS were harmonized, and the individual study GWAS results were subjected to quality control using the EasyQC package in R^36^. To check for study-specific analytical issues, *P*–*Z* plots were generated comparing the beta-estimates (*z*-statistic) to reported *P* values. Effect allele frequency plots were used to identify strand issues, allele miscoding or individuals whose assigned ancestry did not match their genetic ancestry based on effect allele frequencies against a reference set. SE-N plots (inverse of the median standard error versus the square root of the sample size) were also generated to identify any improper transformations of the traits, incorrect use of the regression model or unit errors. The consistency of some known associations for hypothyroidism was also checked in each study as a positive control.

Variants with a MAF ≤ 0.5%, an implausible |beta| ≥ 1,000 or a poor imputation quality score of ≤0.4 were excluded before the meta-analyses were conducted. Two independent analysts conducted the multi-ancestry, sex-specific and European ancestry-specific meta-analyses using the inverse variance-weighting and fixed-effect model approach from the METAL software^21^. Only variants with a MAF > 1% and a sample size for association of at least 75% of the total (or sex/ethnicity stratified) GWAS meta-analysis sample size were considered for further analyses. A pre-specified genome-wide significance threshold was applied at *P* < 5 × 10^−8^. The *I*^2^ statistic was used to assess between-study heterogeneity, and residual inflation of the meta-analysis results was assessed using the LDSC regression intercept^50^.

To identify independently associated variants within each locus, LD-based clumping was performed using PLINK. The HRC imputed UK Biobank data served as a reference dataset, as previously described^49^. In brief, variants with an LD (*r*^2^) of ≤0.01 within windows of ±1 Mb were retained. This LD-based clumping procedure ensured the selection of variants relatively independent of each other, thereby reducing redundancy in the data. Significantly associated independent variants with a distance of less than 1 Mb were combined into a single locus. Variants were classified as known if they were correlated (*r*^2^ > 0.1) with a previously known variant within a ±10 Mb distance^17,22,23^. Loci were deemed known if they included a known variant. This comprehensive approach, leveraging PLINK’s LD pruning strategy, facilitated the identification of independent loci associated with the trait of interest while considering LD and known variants^49^.

Fine-mapping of the results to distinguish between causal candidate variants and other variants in LD in the associated loci was performed with SuSiEx^33^, using both the multi-ancestry and the European ancestry GWAS results as input with the 1000 Genomes (v3) and the UK Biobank-based reference panel described below for LD estimation, respectively. The maximum number of causal signals in the model was set to ten. The fine-mapping results were limited to variants with a posterior inclusion probability of >1%, and only credible sets that included at least one variant with a GWAS association *P* < 1 × 10^−5^ were retained.

### Pathway analysis

The gene list for the associated Hashimoto’s thyroiditis variants as determined by Open Targets Genetics was used as input for the pathway enrichment analyses. We used the matched enrichment approach described previously for the analyses^60^. First, we constructed a reference database of Entrez gene identifiers using the Bioconductor package org.Hs.eg.db (v3.20.0), incorporating gene length, the number of independent SNPs per gene, associated Gene Ontology terms and KEGG pathways.

Next, we conducted 50 million random draws of an equal number of genes as contained in the gene list, matching for deciles of the number of independent SNPs and deciles of gene length. We then compared the overlap of enriched terms between the original list and the random sets to compute empirical *P* values. The analysis was repeated after excluding genes located in the MHC region. To account for multiple testing, only results that passed a Benjamini–Hochberg false discovery rate of <0.05 were reported.

### Functional enrichment and tissue expression analysis

MAGMA software, available in the FUMA platform (v1.3.7), was used to conduct gene-set and tissue-enrichment analyses for 54 tissue types, using GTEx (v8) RNA-seq data. Further details regarding the specific analytic methods can be found elsewhere^38^. To identify independent variants, *r*^2^ = 0.05 was used (the lowest option in FUMA).

DEPICT (v1 rel194) was used as a complementary tissue and cell enrichment method to identify cell and tissue enrichment, based on gene assignment within DEPICT. Additional details regarding this method can be found in previous publications^61^.

To identify cell types enriched for Hashimoto’s thyroiditis, LDSC-SEG analysis using gene expression data was performed^62^. LDSC-SEG performs a regression analysis from the full summary statistics combined with LD information, regressing SNP-specific effect sizes on LD score-derived weights to quantify the proportion of phenotypic variance explained by additive genetic factors. The LDSC coefficient value is characterized by the increase in heritability per SNP associated with the given cell type conditional on all cell types. Enriched heritability as measured by the coefficient *P* value implies that the Hashimoto’s thyroiditis-associated variants exhibit a higher-than-expected contribution to the variance in specifically expressed genes in defined cell types. The *t*-statistics for each gene expressed in a particular cell type were estimated, then all genes were ranked according to the *t*-statistics, and the top 10% of genes exhibiting the most elevated *t*-statistics defined the gene set corresponding to each cell type. Next, the partitioned heritability was estimated for each gene set using Hashimoto’s thyroiditis GWAS as input. Finally, the coefficient *P* value was calculated from the coefficient *z*-score and tests whether the coefficient is significantly positive, which was used to assess enrichment. We applied the Bonferroni correction to account for testing multiple cell types. This method was applied to data from scRNA-seq of two thyroids from a previous study and separately on expression data from the DICE dataset (Supplementary Tables 13 and 14)^41,43^. The thyroid scRNA-seq data were downloaded from the Gene Expression Omnibus under accession number GSE182416 and processed in the R package Seurat. For these data, we performed the analysis first on the whole 54,726 cells in eight broad cell types and then on the subset of 23,758 cells in 11 immune cell types. The scRNA-seq information for analyzing the combined dataset of two patients with Hashimoto’s thyroiditis and one control was obtained using published data from eight cell types from thyroid glands^63^. The DICE expression data were downloaded as transcripts per million from the DICE Database (https://dice-database.org). The 10% specifically expressed genes were deduced for each cell type in each dataset as previously described^50,62^. The comprehensive analysis procedure for LDSC-SEG is available online (https://github.com/bulik/ldsc/wiki/Cell-type-specific-analyses). Differentially expressed genes between the scRNA-seq Hashimoto’s thyroiditis and control dataset were revealed using the R package Seurat, with associations with an adjusted *P* < 0.05 as significant.

### Colocalization of hypothyroid and *cis*-eQTL associations

To conduct the colocalization analyses, version 2.1.6 of the ‘gtx’ R package (https://github.com/tobyjohnson/gtx) was used, which includes Giambartolomei’s colocalization method with modifications. The analysis aimed to identify tissue–gene pairs that featured reported eQTLs within a 100 kb range of each GWAS index variant for every locus. To determine significant outcomes for all colocalization analyses, a posterior probability of at least 0.85 for the H4 test was used. The H4 test probability is a measure of the accuracy of the assumed model, evaluating the probability that the trait and expression data are associated and share a single causal variant^64^. Final results were filtered for protein-coding genes according to the gene ID using the R package ‘biomaRt’.

### Genetic correlation with other traits

To explore the potential for co-regulation or a shared genetic basis, we assessed the genome-wide genetic correlations between Hashimoto’s thyroiditis and five autoimmune diseases (rheumatoid arthritis, vitiligo, Sjögren syndrome, celiac disease and psoriatic arthritis), thyroid function traits (TSH, free T4, free T3 and total T3) and TPOAb status^19,44–50^. Using LDSC^50^, which accommodates overlapping samples, we used GWAS summary statistics as the input to determine pairwise genetic correlations between Hashimoto’s thyroiditis and each of the GWAS summary statistics for thyroid function traits and the top five comorbid autoimmune diseases; additional detail can be found in Supplementary Table 16. A Bonferroni-corrected level of significance (*P* = 0.05 / 10 = 0.005) was used to evaluate statistical significance.

### PheWAS

To explore the phenome-wide effect of the genomic variants uncovered in the GWAS, we performed PheWAS analyses using a polygenic score based on the 155 significant index variants using the 181,899 individuals in the *All of Us* Research Program Curated Data Repository (v7) that had demographic survey data, electronic health record (EHR) data and genome sequencing at the time of analysis. *All of Us* focuses on recruiting individuals underrepresented in biomedical research. In the program, EHR data were collected from various enrolling sites and standardized with the Observational Medical Outcomes Partnership (OMOP) common data model^65^. Whole-genome sequencing was processed as described previously^65^. The polygenic score was created using the variants, effect alleles and allelic effect size from those found in the meta-analysis. This score was defined as a sum of the number of effect alleles an individual had in their genome, weighted by the Hashimoto’s thyroiditis allele effect size. Each participant’s score was adjusted by the average difference in the polygenic score density curves between individuals with versus without 2+ Hashimoto’s thyroiditis codes. This step was performed to ensure a clear divide between Hashimoto’s thyroiditis cases versus controls. We used these adjusted scores as the independent variable for the PheWAS, using PhecodeX 1.0 to map ICD9 and ICD10 codes to conditions and diseases^66^. We required each Phecode to have at least 50 cases and 50 controls to be analyzed. PheWAS betas were adjusted by ten principal components, sex at birth, age at last diagnostic event, number of diagnosis codes, number of unique diagnosis codes and the number of days between start and end dates for the diagnosis code in the EHR data. Phecodes with *P* values above the Bonferroni correction (*P* = 0.05 / 1,422 = 3.5 × 10^−5^) were deemed significant. Phecodes for Hashimoto’s thyroiditis or hypothyroidism were removed, as individuals with Hashimoto’s thyroiditis from *All of Us* were also included in the cases used in the GWAS meta-analysis (and resulting in highly significant *P* value associations).

### Mendelian randomization

The causal effects of autoimmune hypothyroidism on different traits were assessed by performing two-sample MR on HDL cholesterol, LDL cholesterol, myocardial infarction, non-alcoholic fatty liver disease, waist circumference, diastolic blood pressure, systolic blood pressure, type 2 diabetes, atrial fibrillation, ischemic stroke, sleep apnea syndrome, chronic heart failure, essential hypertension, total triglycerides, body mass index, heart rate, thyroid cancer, Alzheimer’s disease, major depression, anxiety or panic attack, TSH, free T4, free T3 and total T3.

Calculations were performed using the R package TwoSampleMR (https://mrcieu.github.io/TwoSampleMR), with default options for data preprocessing and variant harmonization but without additional LD filtering^52^. Summary statistics were pulled from studies within the R package, and genome-wide significant (*P* < 5 × 10^−8^) independent associations were selected as instruments. Details on the included studies can be found in Supplementary Table 18, and on the instruments in Supplementary Figure 3. Significance was set to *P* < 0.00208 in the inverse variance-weighted MR after correcting for the 24 outcomes tested, and to 0.05 for the sensitivity analysis (weighted median MR). The degree of heterogeneity among the instruments was estimated by the Cochrane *Q* value. Details of these methods can be found in the original publication^52^.

### Gene–drug interaction analysis

The Drug–Gene Interaction Database (DGIdb v5.0.8; https://dgidb.org) was queried to identify known drug interactions with the 105 genes highlighted by our colocalization analysis, of which 23 were found to have drug interactions (Supplementary Table 19). Drugs showing an interaction score—that is, the numeric representation of publication count, source count, the ratio of average known gene partners for all drugs to the known partners for the given drug and the ratio of average known drug partners for all genes to the known partners for the given gene—of >1.0 were selected for a total of 100 drug interactions. An interaction score cutoff of 1 was used to filter out drugs with a lower degree of specificity in the interaction between the drug and gene.

### Reporting summary

Further information on research design is available in the Nature Portfolio Reporting Summary linked to this article.

## Online content

Any methods, additional references, Nature Portfolio reporting summaries, source data, extended data, supplementary information, acknowledgements, peer review information; details of author contributions and competing interests; and statements of data and code availability are available at 10.1038/s41588-026-02704-w.

## Supplementary information

Supplementary Information Supplementary Notes 1–3 and Supplementary Figs. 1–3.

Reporting Summary

Peer Review File

Supplementary Table Supplementary Tables 1–19.

## Source data

Source Data Extended Data Figs. 4 and 7 Statistical source Data for Extended Data Figs. 4 and 7.

## Data Availability

The individual participant data included in this project are not publicly available owing to data privacy laws but can be obtained by applying to the individual studies on request. Data from the UK Biobank can be requested directly through the study website (https://www.ukbiobank.ac.uk). Publicly available data from the GTEx Project version 8 release were used (https://gtexportal.org), as well as Gene Expression Omnibus (accession number GSE182416), the UCSC Genome Browser (https://genome-euro.ucsc.edu), Open Targets Genetics (https://genetics.opentargets.org), the GWAS Catalog (https://www.ebi.ac.uk/gwas) and summary statistics of the following GWAS: HDL cholesterol, LDL cholesterol, myocardial infarction, non-alcoholic fatty liver disease, waist circumference, diastolic blood pressure, systolic blood pressure, type 2 diabetes, atrial fibrillation, ischemic stroke, sleep apnea syndrome, chronic heart failure, essential hypertension, total triglycerides, body mass index, heart rate, thyroid cancer, Alzheimer’s disease, major depression, anxiety or panic attack, TSH, free T4, free T3 and total T3 (Methods). The summary statistics from the GWAS meta-analyses are available on the ThyroidOmics Consortium website (http://www.thyroidomics.com). Source data are provided with this paper.

## Code availability

Unless otherwise specified, the GWAS quality control, post-processing and analysis were carried out using Linux shell, Perl (v5), Python (v2.7) and R (v4.3) with the following packages: gtx, ggplot2, PheWAS, biomaRt, Seurat, org.Hs.eg.db and EasyQC. Additional analysis software included LDSC (v1.0.1) (https://github.com/bulik/ldsc), FUMA (accessed: 11/08/2023) (https://fuma.ctglab.nl/), METAL (release 02-12-2018) (www.sph.umich.edu/csg/abecasis/metal/), PLINK (v1.90b7) (https://www.cog-genomics.org/plink2/), DEPICT (v1_rel194) (https://github.com/perslab/depict), Two Sample MR (v0.5.9) (https://mrcieu.github.io/TwoSampleMR/), Drug–Gene Interaction Database (accessed: 03/30/2025) (https://dgidb.org), LDSC-SEG (v1.0.1) (https://github.com/bulik/ldsc/wiki/Cell-type-specific-analyses) and HESS (v0.5; release 11/09/2017) (https://huwenboshi.github.io/hess-0.5/).
