# Supplementary material for: Multi-ancestry genome-wide association analyses provide insights into the genetic basis of Hashimoto’s thyroiditis
Source: Nat Genet. 2026 Jul 29;58(8):1855–65. doi: 10.1038/s41588-026-02704-w (PMC13447099; doi:10.1038/s41588-026-02704-w)
Supplement: Supplementary file 1 [file 41588_2026_2704_MOESM1_ESM.pdf]

nature genetics

Supplementary information

https://doi.org/10.1038/s41588-026-02704-w

Multi-ancestry genome-wide association analyses provide insights into the genetic basis of Hashimoto's thyroiditis

In the format provided by the authors and unedited

Table of Contents

Supplementary Note 1: The Biobank Japan Project 2

Supplementary Note 2: Drug target analysis results 3

Supplementary Note 3: Study specific acknowledgements 3

- *All of Us* 3
- Biobank Japan 4
- CROHT 4
- DiscovEHR 4
- Estonian Biobank 4
- GoDARTS-SHARE 4
- BMC (Latvia) 5
- HUNT 5
- MGI 5

Supplementary Figure 1: Quantile-quantile plots for all Hashimoto's thyroiditis GWAS meta-analyses 6

Supplementary Figure 2. Regional association plots of the *CAPZB* locus 7

Supplementary Figure 3. Mendelian randomization plots of the significant results 8

Supplementary Note 1: The Biobank Japan Project

Koichi Matsuda[38,39], Yuji Yamanashi[40], Yoichi Furukawa[41], Takayuki Morisaki[42], Yukinori Okada[14,23,24,25,43], Yoshinori Murakami[44], Yoichiro Kamatani[45], Kaori Muto[46], Akiko Nagai[39], Yusuke Nakamura[47], Wataru Obara[48], Ken Yamaji[49], Kazuhisa Takahashi[50], Satoshi Asai[51,52], Yasuo Takahashi[52], Shinichi Higashiue[53], Shuzo Kobayashi[53], Hiroki Yamaguchi[54], Yasunobu Nagata[54], Satoshi Wakita[54], Chikako Nito[55], Yu-ki Iwasaki[56], Shigeo Murayama[57], Kozo Yoshimori[58], Yoshio Miki[59], Daisuke Obata[60], Masahiko Higashiyama[61], Akihide Masumoto[62], Yoshinobu Koga[62] & Yukihiro Koretsune[63]

38. Laboratory of Genome Technology, Human Genome Center, Institute of Medical Science, The University of Tokyo, Tokyo, Japan.
39. Laboratory of Clinical Genome Sequencing, Graduate School of Frontier Sciences, The University of Tokyo, Tokyo, Japan.
40. Division of Genetics, The Institute of Medical Science, The University of Tokyo, Tokyo, Japan.
41. Division of Clinical Genome Research, Institute of Medical Science, The University of Tokyo, Tokyo, Japan.
42. Department of Computational Biology and Medical Sciences, Graduate School of Frontier Sciences, BioBank Japan, Institute of Medical Science, The University of Tokyo, Tokyo, Japan.
43. Premium Research Institute for Human Metaverse Medicine (WPI-PRIMe), Osaka University, Osaka, Japan.
44. Department of Cancer Biology, Institute of Medical Science, The University of Tokyo, Tokyo, Japan.
45. Laboratory of Complex Trait Genomics, Graduate School of Frontier Sciences, The University of Tokyo, Tokyo, Japan.
46. Department of Public Policy, Institute of Medical Science, The University of Tokyo, Tokyo, Japan.
47. The Institute of Medical Science, The University of Tokyo, Tokyo, Japan.
48. Department of Urology, Iwate Medical University, Iwate, Japan.
49. Department of Internal Medicine and Rheumatology, Juntendo University Graduate School of Medicine, Tokyo, Japan.
50. Department of Respiratory Medicine, Juntendo University Graduate School of Medicine, Tokyo, Japan.
51. Division of Pharmacology, Department of Biomedical Science, Nihon University School of Medicine, Tokyo, Japan.
52. Division of Genomic Epidemiology and Clinical Trials, Clinical Trials Research Center, Nihon University. School of Medicine, Tokyo, Japan.
53. Tokushukai Group, Tokyo, Japan.
54. Departmentof Hematology, Nippon Medical School, Tokyo, Japan.
55. Laboratory for Clinical Research, Collaborative Research Center, Nippon Medical School, Tokyo, Japan.
56. Department of Cardiovascular Medicine, Nippon Medical School, Tokyo, Japan.

57. Tokyo Metropolitan Geriatric Hospital and Institute of Gerontology, Tokyo, Japan.
58. Fukujuji Hospital, Japan Anti-Tuberculosis Association, Tokyo, Japan.
59. The Cancer Institute Hospital of the Japanese Foundation for Cancer Research, Tokyo, Japan.
60. Center for Clinical Research and Advanced Medicine, Shiga University of Medical Science, Shiga, Japan.
61. Department of General Thoracic Surgery, Osaka International Cancer Institute, Osaka, Japan.
62. Iizuka Hospital, Fukuoka, Japan.
63. National Hospital Organization Osaka National Hospital, Osaka, Japan.

Supplementary Note 2: Drug target analysis results

The Drug-Gene Interaction Database (DGIdb) was queried, using a list of unique genes derived from the colocalization analysis, to investigate whether the HT-associated genes encode proteins that are predicted targets for approved drugs and those in clinical trial testing[52]. Of the 105 unique candidate causal genes, DGIdb identified 101 known interactions for 23 genes with currently available therapeutics with a mean interaction score of 2.82 (Supplementary Table 19). The interaction score serves as a measure of the specificity of a drug's action on its target; for example, if a drug has many targets, it is considered less precise and, therefore, has a low interaction score. The gene with the highest interaction score is *DOCK10* (Binetrakin, 20.88), which is currently not a FDA-approved drug but is studied in clinical trials. Binetrakin is a recombinant interleukin-4 (IL-4) that can bind to IL-4 receptors on cell surfaces, promoting the growth and differentiation of activated B-cells and T-cells. However, this would likely promote an increased immune response in HT. Binetrakin is currently being tested in clinical trials to treat a wide range of diseases including asthma, allergic rhinitis, melanoma, and HIV (https://clinicaltrials.gov). The drugs identified may serve as candidates for functional studies, as there currently is no treatment for the immune response underlying HT, only for the resulting hypothyroidism (i.e. T4 replacement therapy).

Supplementary Note 3: Study specific acknowledgements

All of Us

The *All of Us* Research Program would not be possible without the partnership of its participants. We gratefully acknowledge *All of Us* participants for their contributions, without whom this research would not have been possible. We also thank the National Institutes of Health's *All of Us Research Program* for making available the participant data examined in this study. The *All of Us* Research Program is supported by the National Institutes of Health, Office of the Director: Regional Medical Centers (1 OT2 OD026549; 1 OT2 OD026554; 1 OT2 OD026557; 1 OT2 OD026556; 1 OT2 OD026550; 1 OT2 OD 026552; 1 OT2 OD026553; 1 OT2 OD026548; 1 OT2 OD026551; 1 OT2 OD026555; IAA: AOD21037, AOD22003, AOD16037, AOD21041), Federally

Qualified Health Centers (HHSN 263201600085U), Data and Research Center (5 U2C OD023196), Biobank (1 U24 OD023121), The Participant Center (U24 OD023176), Participant Technology Systems Center (1 U24 OD023163), Communications and Engagement (3 OT2 OD023205; 3 OT2 OD023206) and Community Partners (1 OT2 OD025277; 3 OT2 OD025315; 1 OT2 OD025337; 1 OT2 OD025276). This work was supported by the National Human Genome Research Institute intramural research program, grant number: ZIA HG200417. The contributions of the NIH authors are considered Works of the United States Government. The findings and conclusions presented in this paper are those of the authors and do not necessarily reflect the views of the NIH or the U.S. Department of Health and Human Services.

Biobank Japan

The BioBank Japan was supported by the Tailor-Made Medical Treatment program (the BioBank Japan Project) of the Ministry of Education, Culture, Sports, Science, and Technology (MEXT), the Japan Agency for Medical Research and Development (AMED).

CROHT

We are grateful to all study participants for their enrollment in this study. We also thank all physicians (prof. Ante Punda, MD, Prof. Veselin Škrabić, MD, Asst. Prof. Ana Barić, MD, Sanda Gračan, MD) for their dedicated work in patient recruitment.
Formation of CRO-HT Biobank was supported by the Croatian Science Foundation (UIP-11-2013-4950). The “10 001 Dalmatians” project was supported by the MRC Human Genetics Unit, The Croatian Ministry of Science, Education and Sports (Grant 216-1080315-0302), the European Union framework program 6 EUROSPAN project (Contract no. LSHGCT-2006-018947), EU FP7 BBMRI-LPC (Biobanking and biomolecular resources research infrastructure—Large prospective cohort, Contract 313010) and the Croatian Science Foundation (Grant nos. 8875 and 1498).

DiscovEHR

The analyses of the DiscovEHR dataset were supported by the Regeneron Genetics Center (Tarrytown, NY, USA).

Estonian Biobank

We want to acknowledge the participants and investigators of the Estonian Biobank research team (Lili Milani, Reedik Mägi, Mari Nelis and Georgi Hudjashov) for data collection, genotyping, QC and imputation. This work was supported by the Estonian Research Council grant TK (TK214). Computations were performed at the High Performance Computing Center, University of Tartu.

GoDARTS-SHARE

We are grateful to all participants in this study, the general practitioners, the Scottish School of Primary Care for their help in recruiting participants, and to the whole team, which includes interviewers, computer and laboratory technicians, clerical workers, research scientists, volunteers, managers, receptionists and nurses. The study complies with the Declaration of Helsinki. Data provision and linkage was carried out by the Health Informatics Centre, University

of Dundee (HIC: https://www.dundee.ac.uk/hic) with analysis of anonymised data performed in an International Organization for Standardisation 27001 – and Scottish Government accredited, secure safe haven. HIC standard operating procedures have been reviewed and approved by the NHS East of Scotland Research Ethics Services, and consent for this study was obtained from the NHS Fife Caldicott Guardian. We would also like to acknowledge NHS Tayside the original data owner. Genomic data QC and analyses were undertaken using High Performance Computing hosted by the School of Life Sciences, University of Dundee https://www.lifesci.dundee.ac.uk/high-performance-computing-0 GoDARTS is funded and supported by the Wellcome Trust, Tenovus Scotland and Diabetes UK grants. SHARE is NHS Scotland Research (NRS) infrastructure initiative and is funded by the Chief Scientists Office of the Scottish Government. Additional Funding and initiation of the spare blood retention at NHS Tayside was supported by the Wellcome Trust Biomedical Resource Award Number 099177/Z/12/Z. Ethics committee approval for GoDARTS was provided Tayside Medical Ethics Committee 053/04. Ethics committee SHARE: East of Scotland NHS REC 13/ES/0020

<u>BMC (Latvia)</u>

The authors acknowledge the Latvian Biomedical Research and Study Centre and the Genome Database of the Latvian Population for providing infrastructure and data.

<u>HUNT</u>

The Trøndelag Health Study (HUNT) (https://doi.org/10.1093/ije/dys095, https://doi.org/10.1093/ije/dyac095, https://doi.org/10.1016/j.xgen.2022.100193) is a collaboration between HUNT Research Centre (Faculty of Medicine and Health Sciences (MH), Norwegian University of Science and Technology – NTNU), Trøndelag County Council, Central Norway Regional Health Authority, and the Norwegian Institute of Public Health. The genotyping in HUNT was financed by the National Institutes of Health; Stiftelsen Kristian Gerhard Jebsen; University of Michigan; the Research Council of Norway; the Liaison Committee for Education, Research and Innovation in Central Norway; and the Joint Research Committee between St Olav's Hospital and the Faculty of Medicine and Health Sciences, NTNU. The genotyping and imputation efforts in HUNT were a collaboration between researchers from the Department of Public Health and Nursing (ISM) (MH, NTNU), and the University of Michigan Medical School and the University of Michigan School of Public Health. The genotyping was performed at the Genomics Core Facility (GCF) (MH, NTNU). The ethics approval number for the current work is: 2015/584 (REK midt).

<u>MGI</u>

The authors acknowledge the Michigan Genomics Initiative participants, Precision Health at the University of Michigan, the University of Michigan Medical School Central Biorepository, the University of Michigan Medical School Data Office for Clinical and Translational Research, and the University of Michigan Advanced Genomics Core for providing data and specimen storage, management, processing, and distribution services, and the Center for Statistical Genetics in the Department of Biostatistics at the School of Public Health for genotype data curation, imputation, and management in support of the research reported in this publication.

Supplementary Figure 1: Quantile-quantile plots for all Hashimoto's thyroiditis GWAS meta-analyses

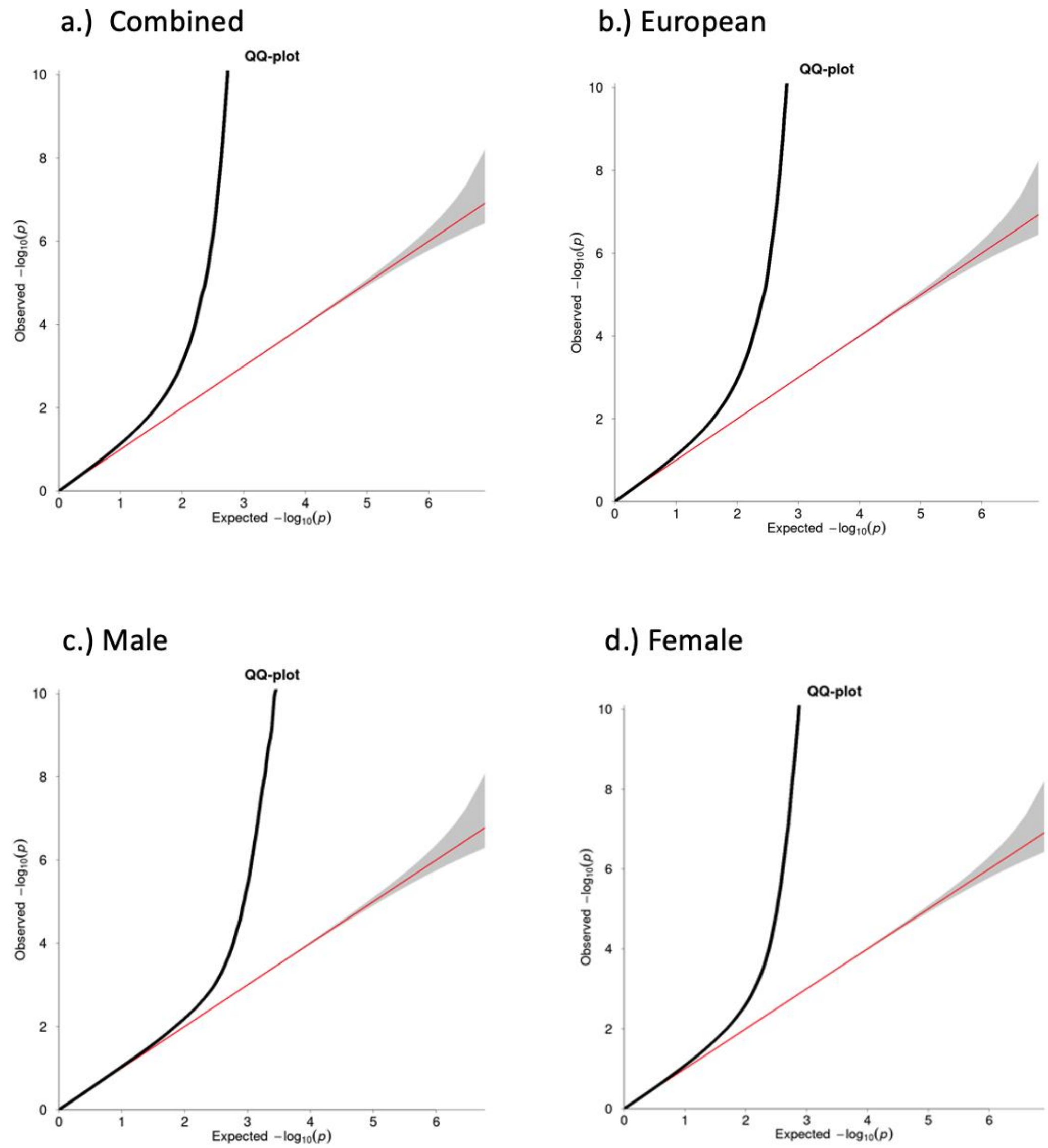

Quantile-quantile (QQ) plots of the GWAS meta-analysis results for a.) combined ancestry, b.) European ancestry, c.) Male, d.) Female. On the y-axis, the observed two-sided p-values of the association test (*z*-statistics) are plotted against the expected distribution under the null hypothesis of no association on the x-axis.

Supplementary Figure 2. Regional association plots of the *CAPZB* locus

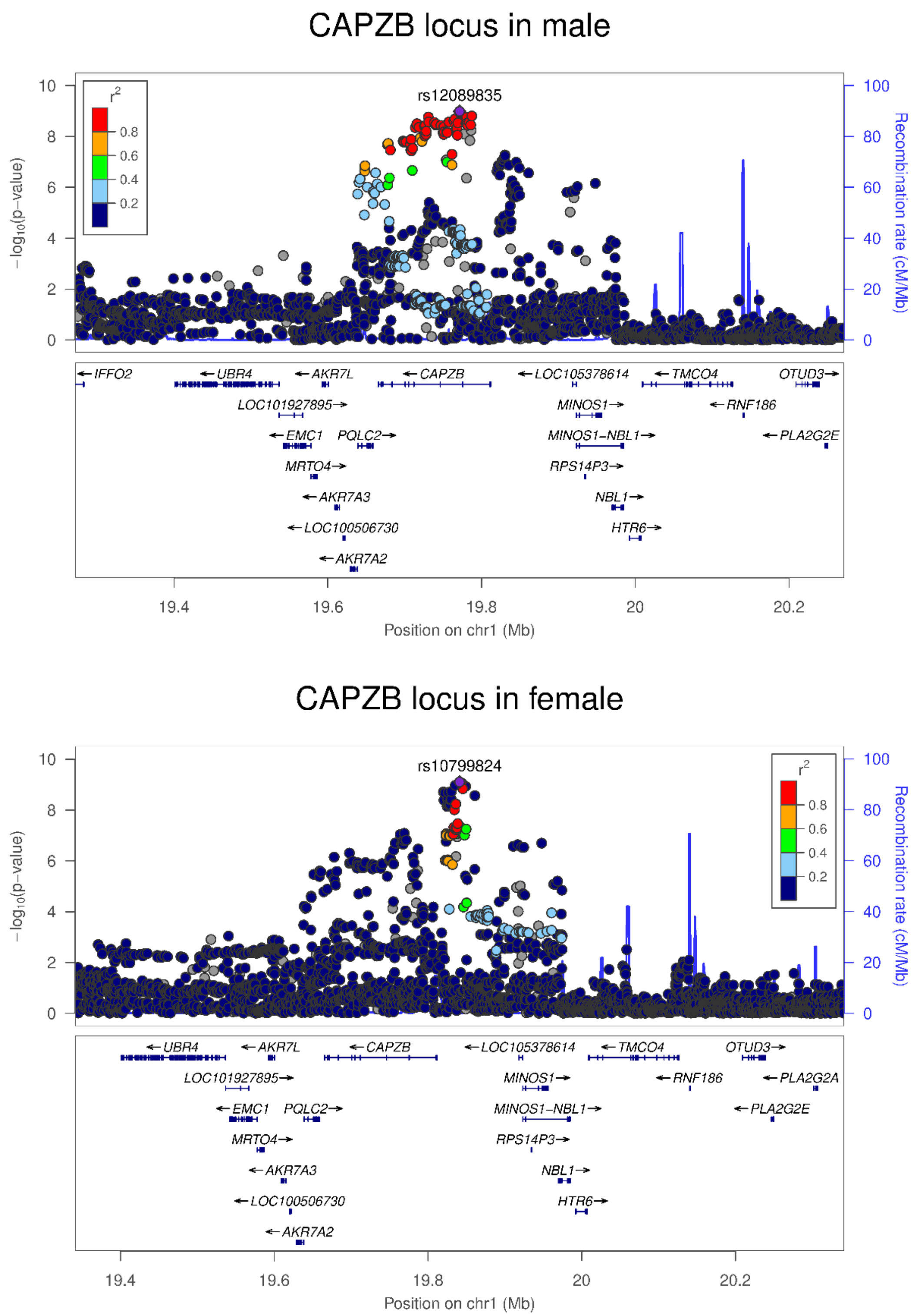

Regional association plots of the *CAPZB* locus of the GWAS meta-analysis in male (top) and female (bottom) stratum with the respective index variant colored in purple and labeled. All p-values were obtained from two-sided association tests (z-statistics).

Supplementary Figure 3. Mendelian randomization plots of the significant results

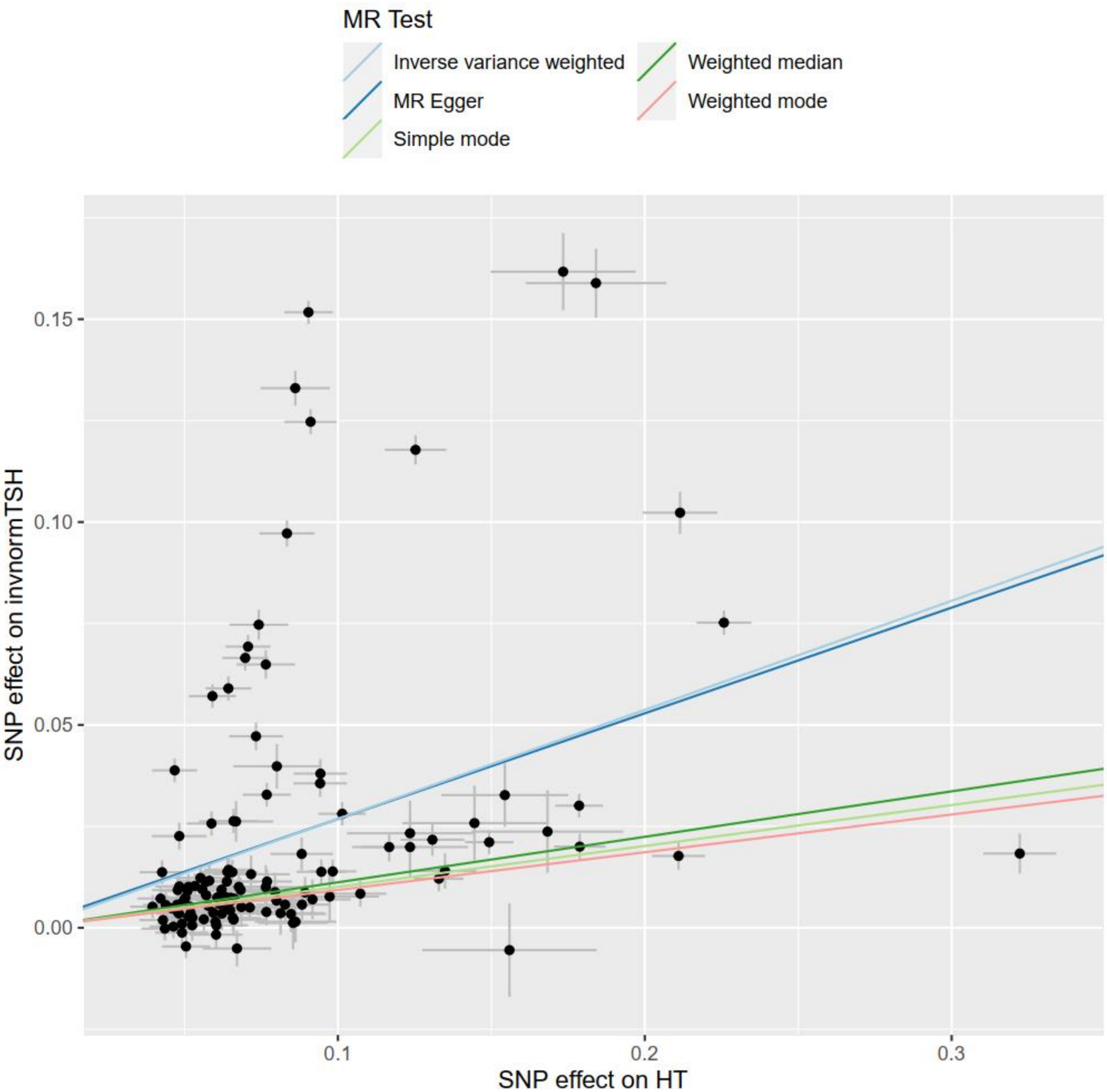

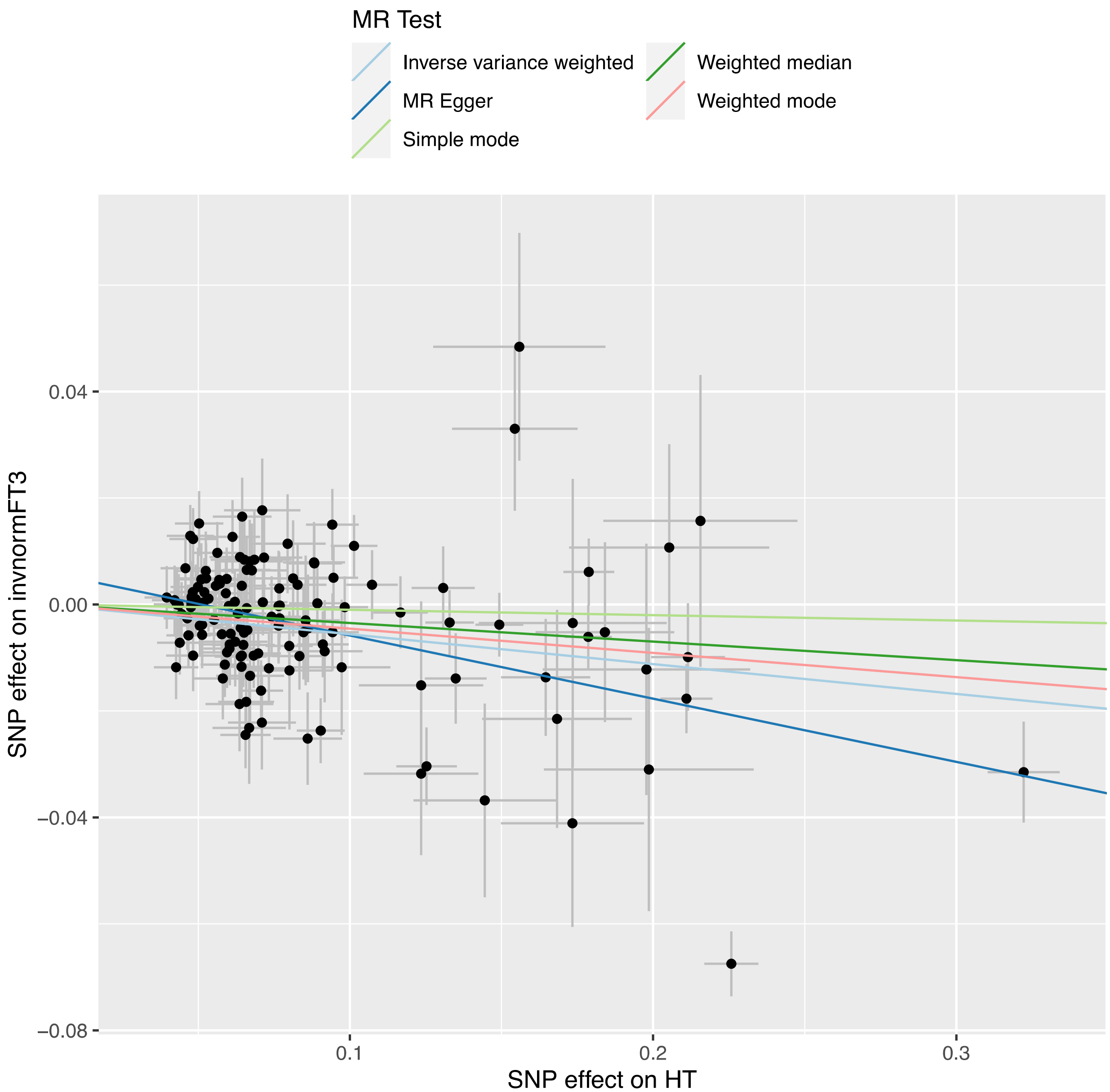

MR Test
Inverse variance weighted
MR Egger
Simple mode
Weighted median
Weighted mode
0.04
0.00
−0.04
−0.08
SNP effect on invnormFT3
0.1
0.2
0.3
SNP effect on HT

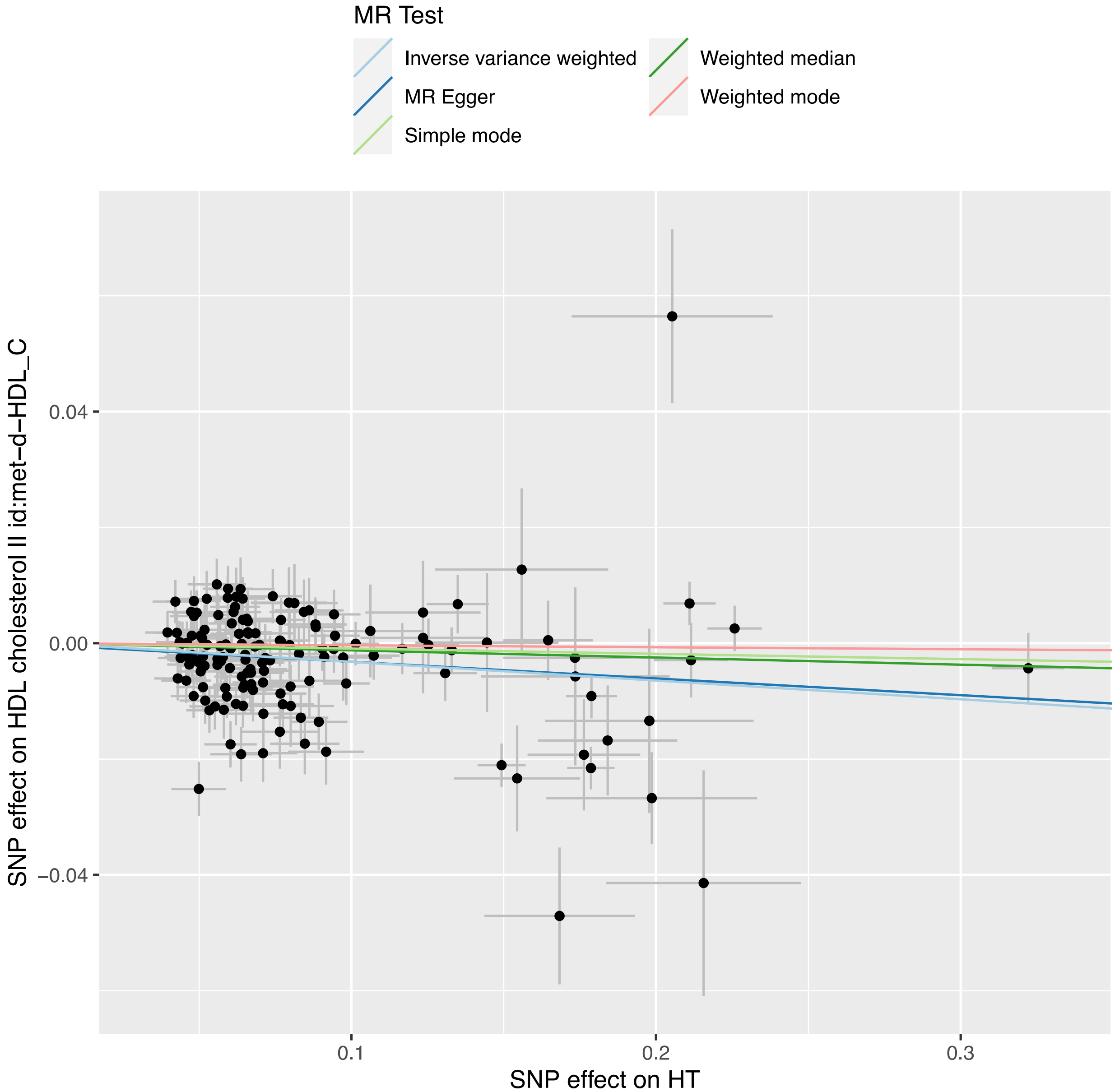
MR Test
Inverse variance weighted
MR Egger
Simple mode
Weighted median
Weighted mode
SNP effect on HDL cholesterol II id:met-d-HDL_C
0.04
0.00
−0.04
0.1
0.2
0.3
SNP effect on HT

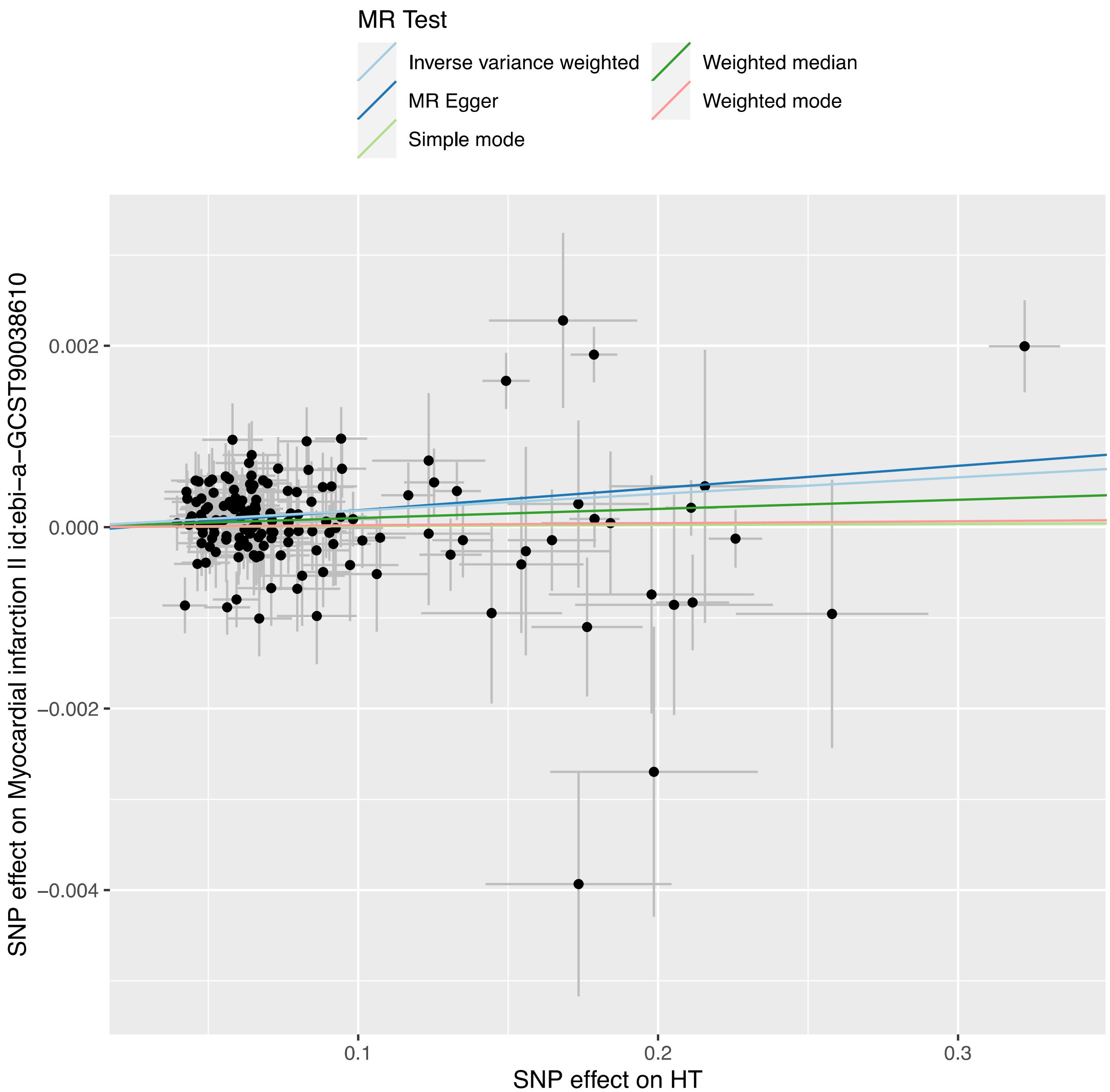
MR Test
Inverse variance weighted
MR Egger
Simple mode
Weighted median
Weighted mode
SNP effect on Myocardial infarction II id:ebi-a-GCST90038610
0.002
0.000
−0.002
−0.004
0.1
0.2
0.3
SNP effect on HT

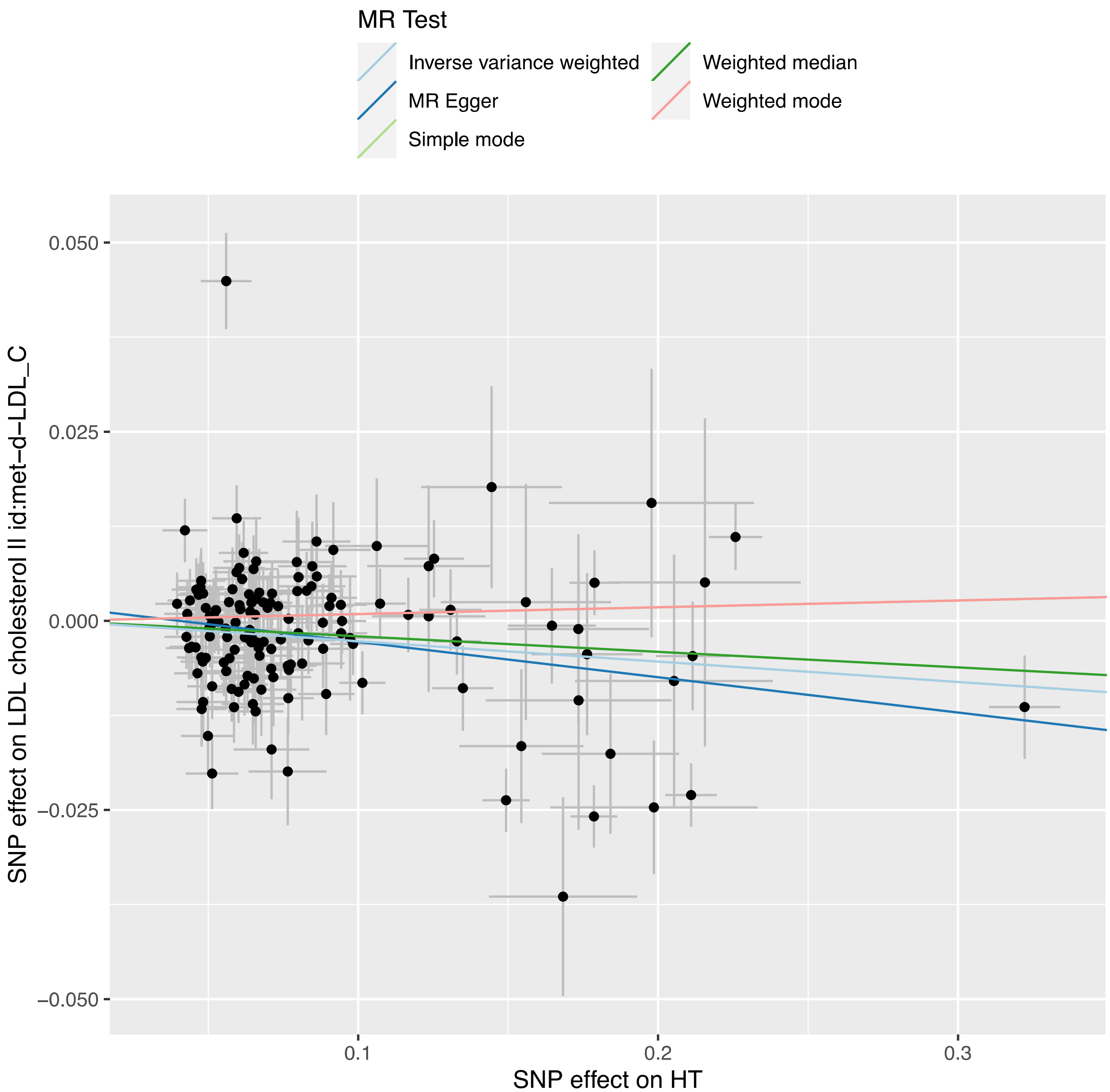
MR Test
Inverse variance weighted
MR Egger
Simple mode
Weighted median
Weighted mode
0.050
0.025
0.000
−0.025
−0.050
0.1
0.2
0.3
SNP effect on HT
SNP effect on LDL cholesterol II id:met-d-LDL_C

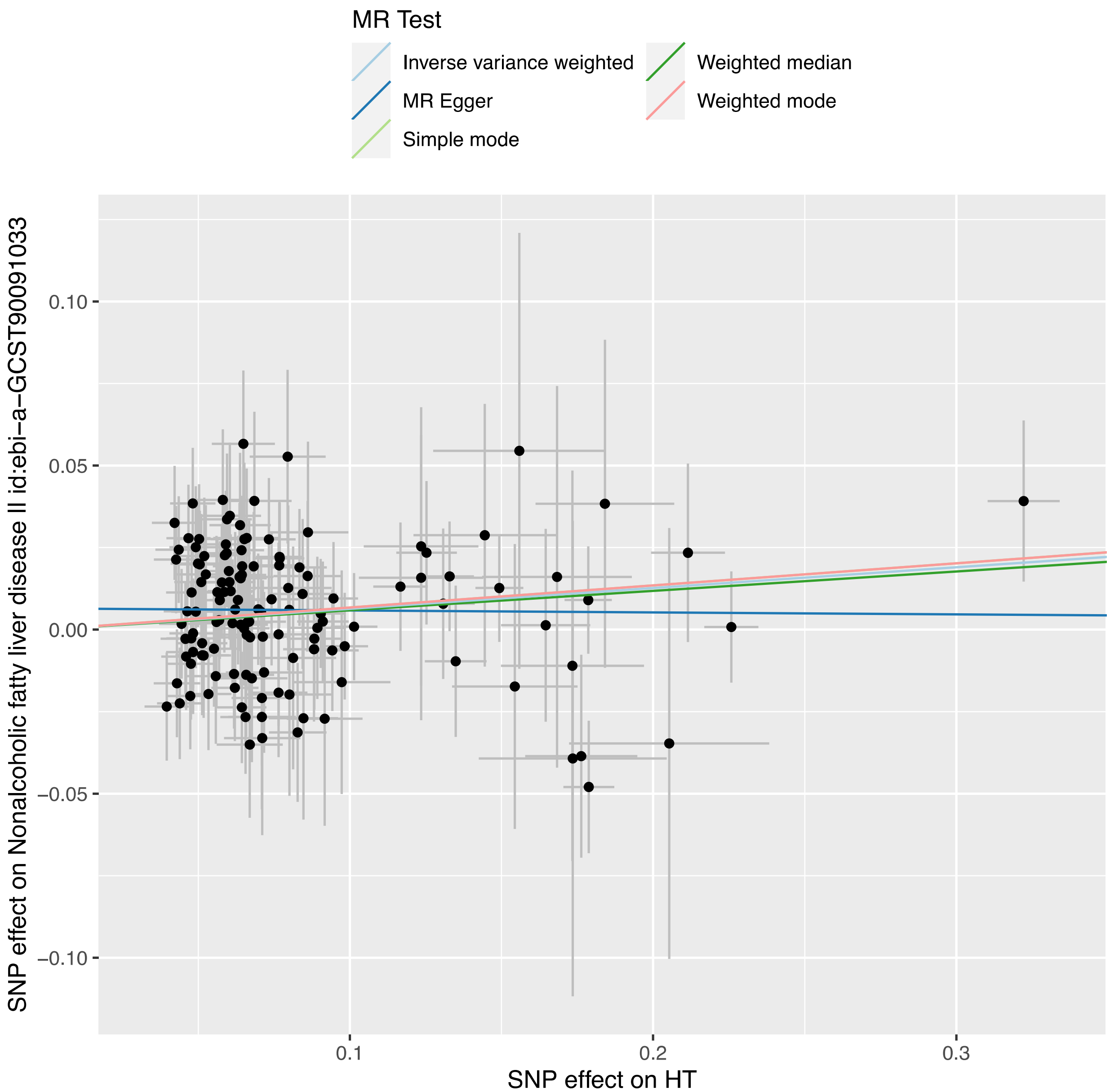
MR Test
Inverse variance weighted
MR Egger
Simple mode
Weighted median
Weighted mode
SNP effect on Nonalcoholic fatty liver disease II id:ebi-a-GCST90091033
0.10
0.05
0.00
−0.05
−0.10
0.1
0.2
0.3
SNP effect on HT

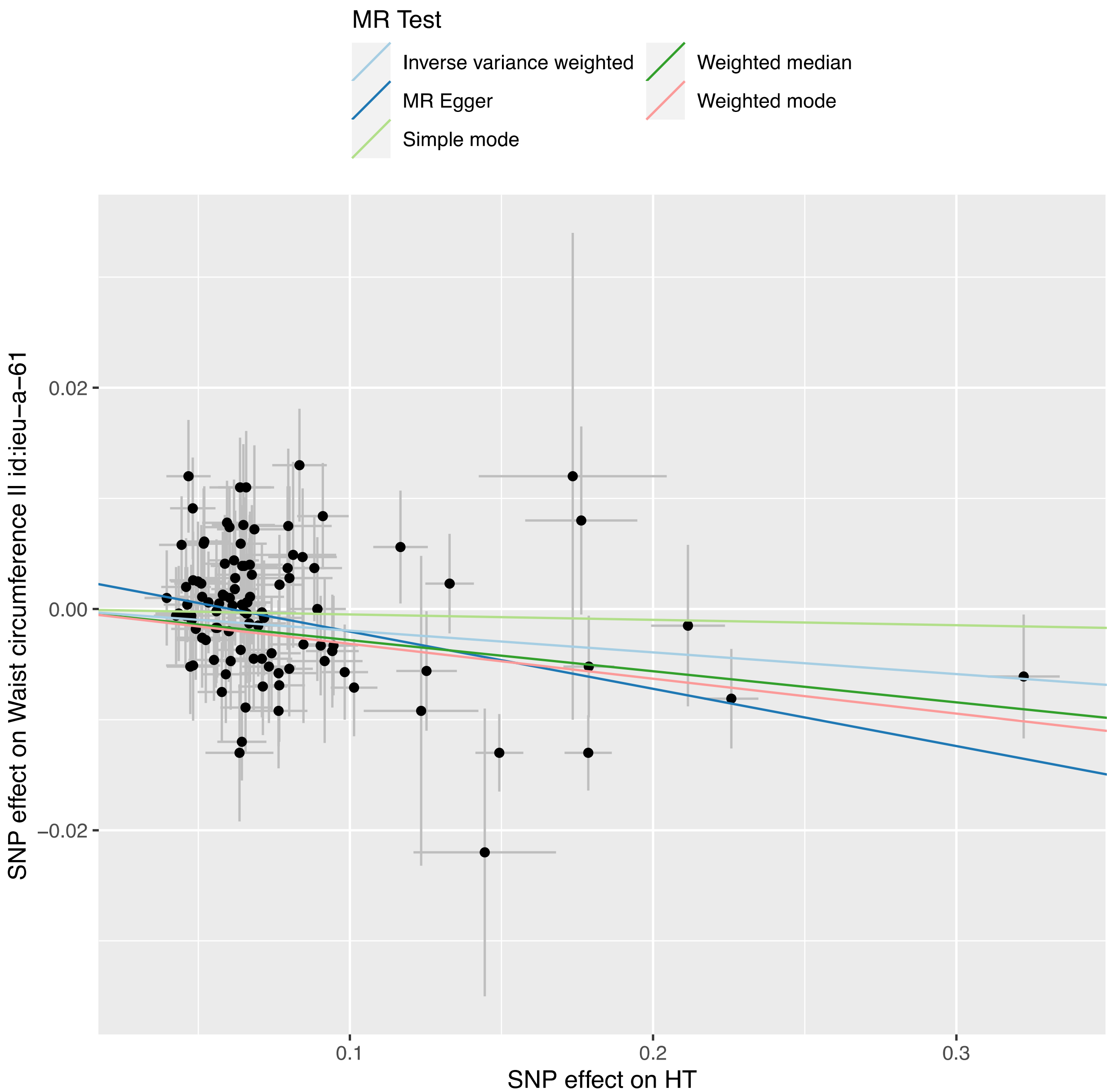
MR Test
Inverse variance weighted
MR Egger
Simple mode
Weighted median
Weighted mode
SNP effect on Waist circumference II id:ieu−a−61
0.02
0.00
−0.02
0.1
0.2
0.3
SNP effect on HT

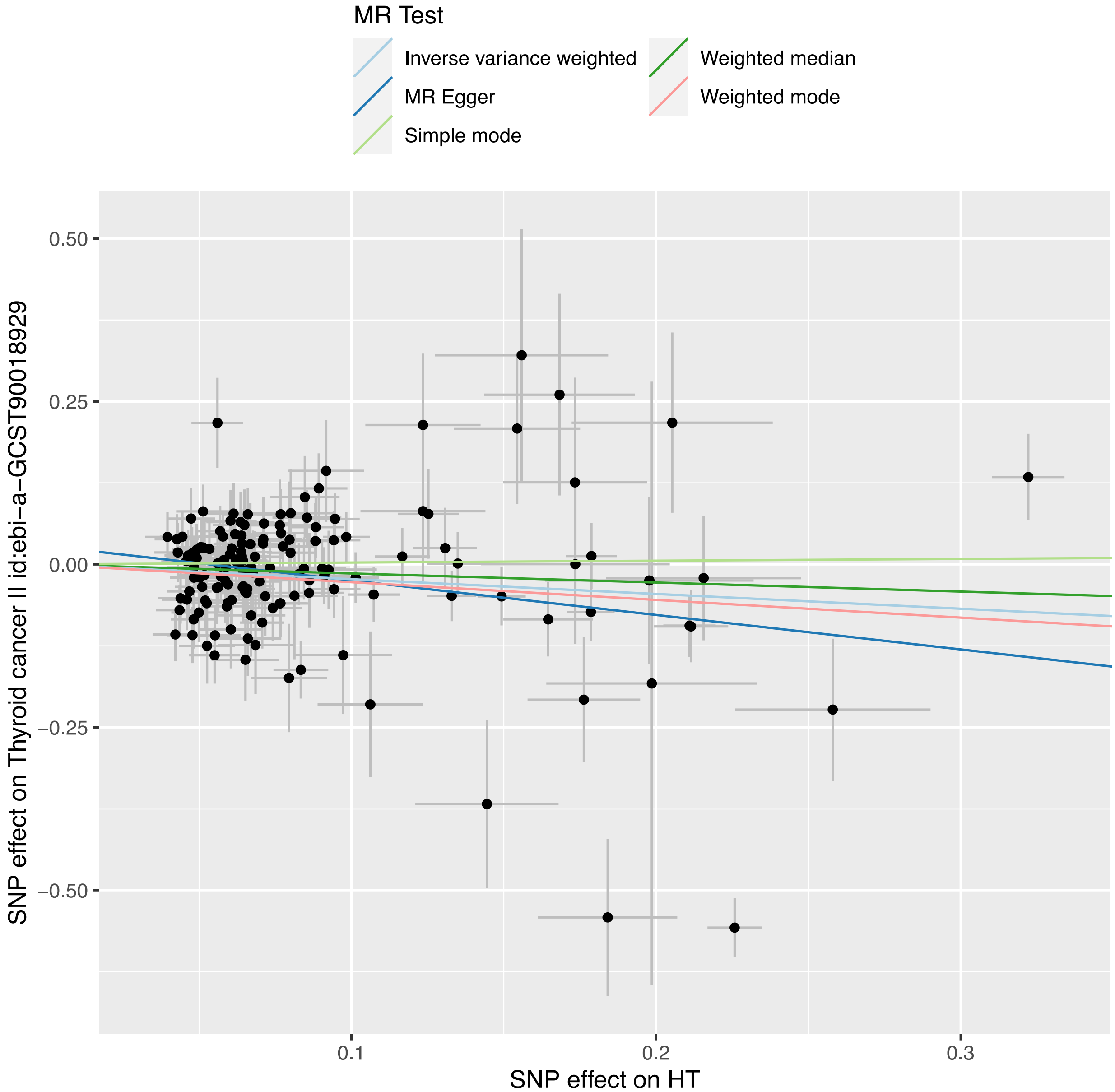
MR Test
Inverse variance weighted
MR Egger
Simple mode
Weighted median
Weighted mode
SNP effect on Thyroid cancer II id:ebi-a-GCST90018929
0.50
0.25
0.00
−0.25
−0.50
0.1
0.2
0.3
SNP effect on HT

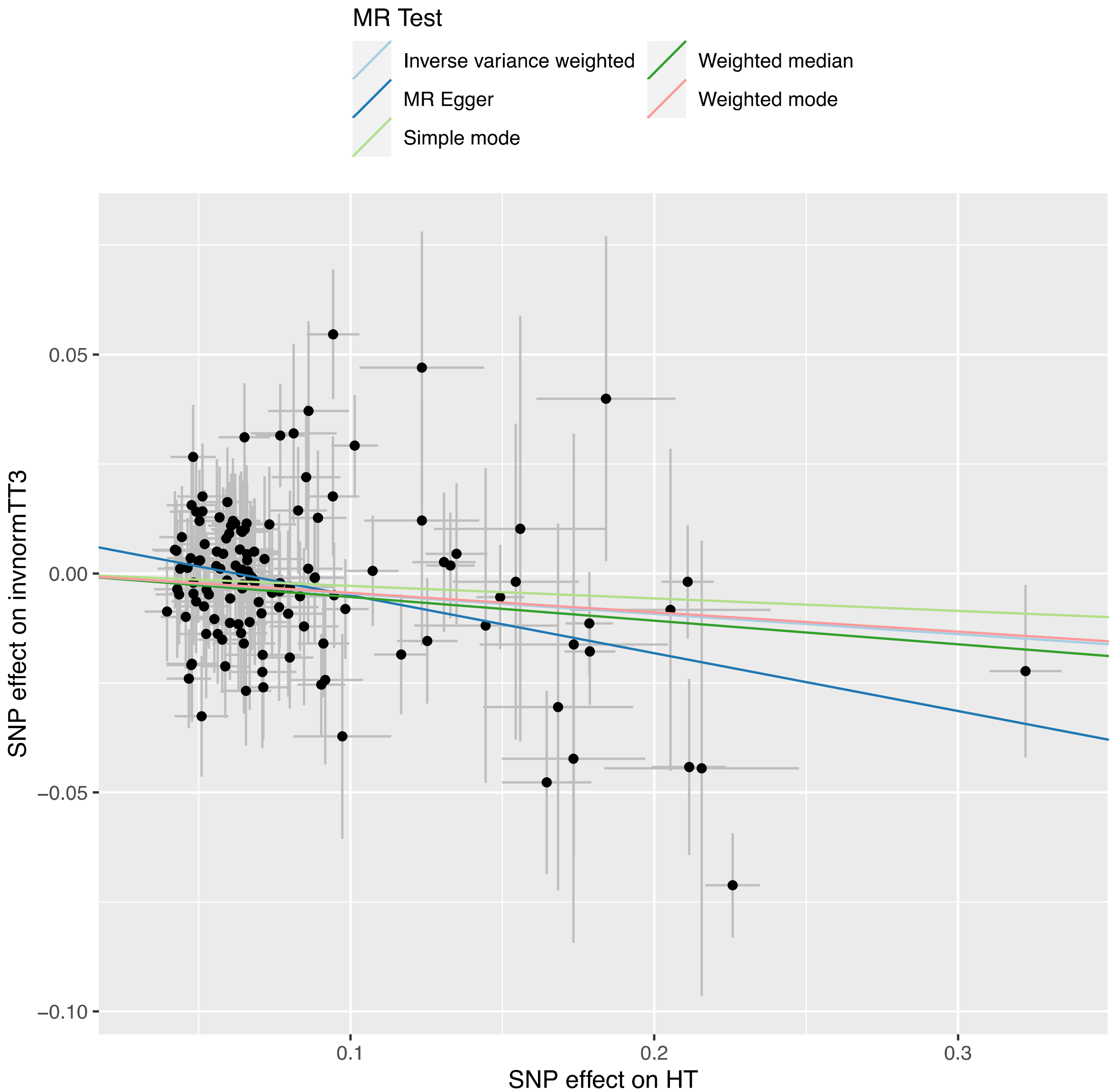
MR Test
Inverse variance weighted
MR Egger
Simple mode
Weighted median
Weighted mode
0.05
0.00
−0.05
−0.10
0.1
0.2
0.3
SNP effect on invnormTT3
SNP effect on HT

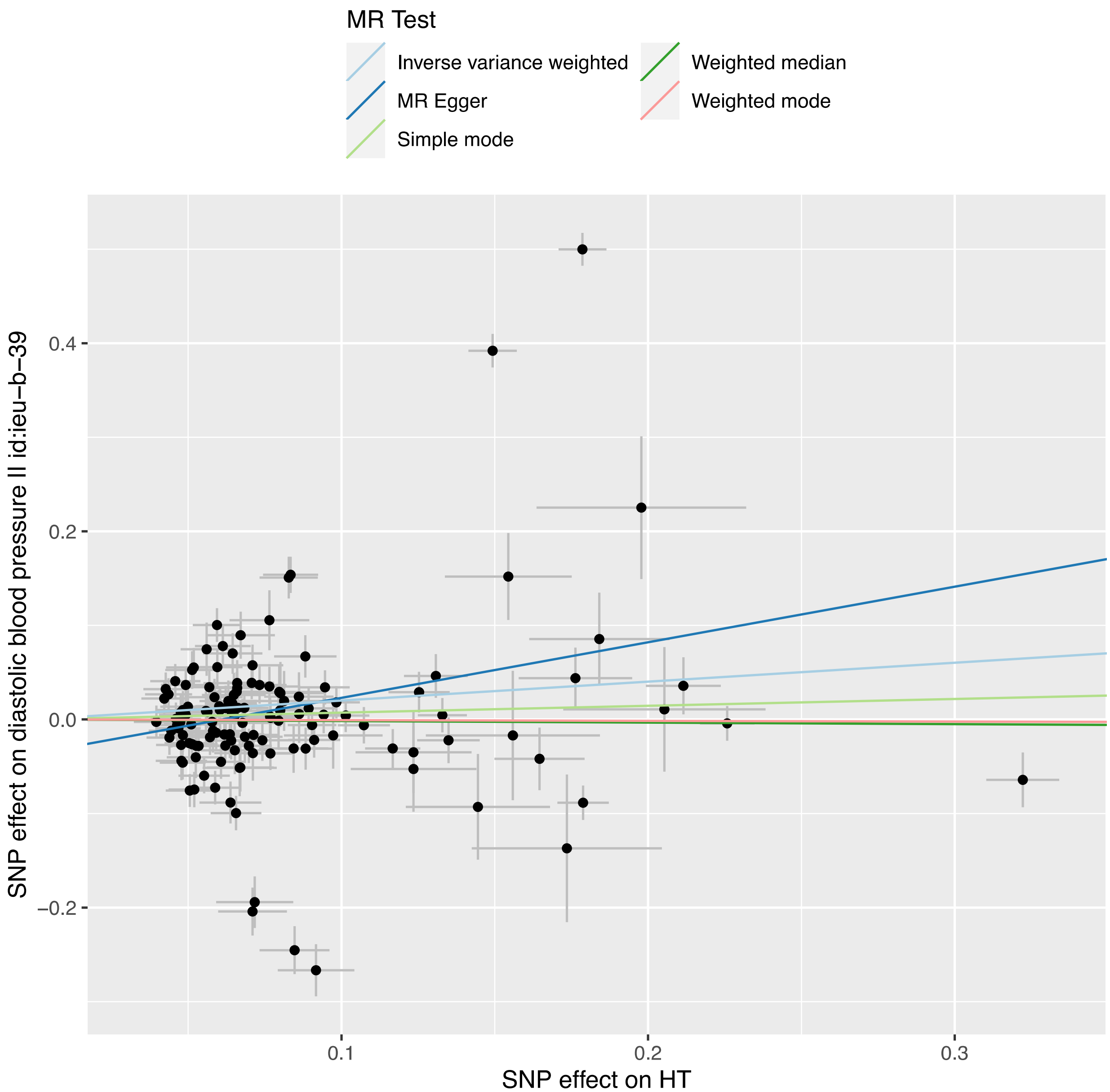

MR Test
Inverse variance weighted
MR Egger
Simple mode
Weighted median
Weighted mode
SNP effect on diastolic blood pressure II id:ieu-b-39
0.4
0.2
0.0
−0.2
0.1
0.2
0.3
SNP effect on HT

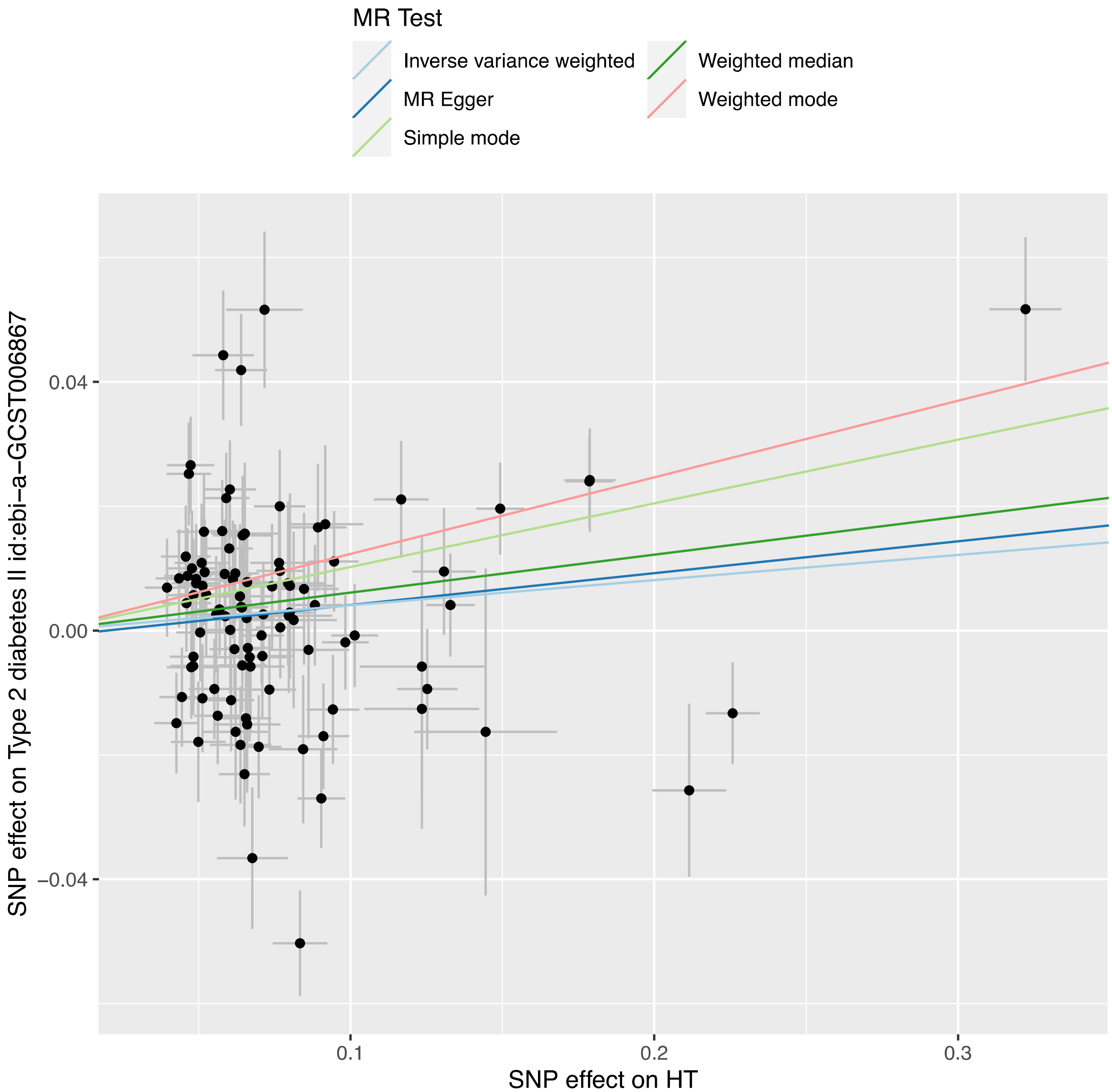

Scatter plot of the effect estimate of each instrumental SNP (dots) on Hashimoto's thyroiditis (HT) (x-axis) vs. the corresponding diseases and traits (y-axis) obtained from the GWAS dataset named in the y-axis description (details are provided in Supplementary Table 18). The error bars represent the standard error (+/- s.e.m.) of the corresponding effect estimate of the SNP. Each line's slope represents the estimated Mendelian randomization (MR) effect per method as stated in the legend.
