# Supplementary material for: Multi-ancestry genome-wide association analyses provide insights into the genetic basis of Hashimoto’s thyroiditis
Source: Nat Genet. 2026 Jul 29;58(8):1855–65. doi: 10.1038/s41588-026-02704-w (PMC13447099; doi:10.1038/s41588-026-02704-w)
Supplement: Supplementary file 2 [file 41588_2026_2704_MOESM2_ESM.pdf]

nature portfolio

Corresponding author(s): Alexander Temer
Marco Medici

Last updated by author(s): Apr 17, 2026

Reporting Summary

Nature Portfolio wishes to improve the reproducibility of the work that we publish. This form provides structure for consistency and transparency in reporting. For further information on Nature Portfolio policies, see our Editorial Policies and the Editorial Policy Checklist.

Statistics

For all statistical analyses, confirm that the following items are present in the figure legend, table legend, main text, or Methods section.

n/a	Confirmed	
☐	☒	The exact sample size (*n*) for each experimental group/condition, given as a discrete number and unit of measurement
☐	☒	A statement on whether measurements were taken from distinct samples or whether the same sample was measured repeatedly
☐	☒	The statistical test(s) used AND whether they are one- or two-sided<br>*Only common tests should be described solely by name; describe more complex techniques in the Methods section.*
☐	☒	A description of all covariates tested
☐	☒	A description of any assumptions or corrections, such as tests of normality and adjustment for multiple comparisons
☐	☒	A full description of the statistical parameters including central tendency (e.g. means) or other basic estimates (e.g. regression coefficient) AND variation (e.g. standard deviation) or associated estimates of uncertainty (e.g. confidence intervals)
☐	☒	For null hypothesis testing, the test statistic (e.g. *F*, *t*, *r*) with confidence intervals, effect sizes, degrees of freedom and *P* value noted<br>*Give P values as exact values whenever suitable.*
☐	☒	For Bayesian analysis, information on the choice of priors and Markov chain Monte Carlo settings
☒	☐	For hierarchical and complex designs, identification of the appropriate level for tests and full reporting of outcomes
☐	☒	Estimates of effect sizes (e.g. Cohen's *d*, Pearson's *r*), indicating how they were calculated

Our web collection on statistics for biologists contains articles on many of the points above.

Software and code

Policy information about availability of computer code

Data collection	No software for data collection for this study was used.
Data analysis	The GWAS quality control, post-processing, and analysis were carried out using Linux shell, Perl v5, Python v2.7, and R v4.3 with the following packages: gtx, ggplot2, PheWAS, biomaRt, Seurat, org.Hs.eg.db, and EasyQC. Additional analysis software included LD Score Regression v1.0.1 (https://github.com/bulik/ldsc), FUMA (accessed: 11/08/2023) (https://fuma.ctglab.nl/), METAL release 02-12-2018 (www.sph.umich.edu/csg/abecasis/metal/), PLINK v1.90b7 (https://www.cog-genomics.org/plink2/), DEPICT v1_rel194 (https://github.com/perslab/depict), Two Sample MR v0.5.9 (https://mrcieu.github.io/TwoSampleMR/), Drug Gene Interaction Database (accessed: 03/30/2025) (https://dgidb.org), LDSC-SEG v1.0.1 (https://github.com/bulik/ldsc/wiki/Cell-type-specific-analyses), and HESS v0.5 (release 11/09/2017) (https://huwenboshi.github.io/hess-0.5/).

For manuscripts utilizing custom algorithms or software that are central to the research but not yet described in published literature, software must be made available to editors and reviewers. We strongly encourage code deposition in a community repository (e.g. GitHub). See the Nature Portfolio guidelines for submitting code & software for further information.

Data

Policy information about availability of data

All manuscripts must include a data availability statement. This statement should provide the following information, where applicable:

- Accession codes, unique identifiers, or web links for publicly available datasets
- A description of any restrictions on data availability
- For clinical datasets or third party data, please ensure that the statement adheres to our policy

The individual participant data included in this project are not publicly available due to data privacy laws but can be obtained by applying to the individual studies on request. Data from the UK Biobank can be requested directly via the study website (https://www.ukbiobank.ac.uk/). Publicly available data from the GTEx Project version 8 release were used (https://gtexportal.org/), the GEO accession number GSE182416 (https://www.ncbi.nlm.nih.gov/geo/query/acc.cgi?acc=GSE182416), the UCSC Genome Browser (https://genome-euro.ucsc.edu/), Open Target Genetics (https://genetics.opentargets.org/), the GWAS Catalog (https://www.ebi.ac.uk/gwas/), as well as summary statistics of the following GWAS: HDL-cholesterol, LDL-cholesterol, myocardial infarction, non-alcoholic fatty liver disease, waist circumference, diastolic blood pressure, systolic blood pressure, type 2 diabetes, atrial fibrillation, ischemic stroke, sleep apnea syndrome, chronic heart failure, essential hypertension, total triglycerides, body mass index, heart rate, thyroid cancer, Alzheimer's disease, major depression, anxiety or panic attack, TSH, free T4, free T3 and total T3 (Methods). The summary statistics from the GWAS meta-analyses are available on the ThyroidOmics Consortium website (http://www.thyroidomics.com).

Research involving human participants, their data, or biological material

Policy information about studies with human participants or human data. See also policy information about sex, gender (identity/presentation), and sexual orientation and race, ethnicity and racism.

Reporting on sex and gender	Sex was determined on self-reported data and/or genetic information. Sex-combined as well as sex-stratified GWAS were performed, and the sex-combined association models were adjusted for sex as a covariate. No individual level data has been shared.
Reporting on race, ethnicity, or other socially relevant groupings	Individuals of European and non-European genetic ancestry were included in this project. Ethnicity was self-reported or estimated based on the genetic profile. Ancestry-specific information was solely used to assess genetic heterogeneity of the GWAS associations. Genetic principal components were included as covariates in the association models where appropriate to adjust for residual genetic ancestry.
Population characteristics	In this project, predominantly population-based studies were included. Details of each cohort are provided in the Supplementary Table 1.
Recruitment	No participants were recruited specifically for this projects. Recruitment of the participants of the individual studies included in this projects are described in the respective study specific publications listed in the Supplementary Table 1.
Ethics oversight	The All of Us Institutional Review Board (IRB); research ethics committees at the Institute of Medical Science, the University of Tokyo, the RIKEN Yokohama Institute; Ethics Committees of the University of Split, School of Medicine and the University Hospital Split; MyCode Governing Board; Estonian Committee on Bioethics and Human Research; NHS Health Research Authority: East of Scotland Research Ethics Service; The Central Medical Ethics Committee of Latvia (CMEC); Mid-Norway Regional Committee for Medical and Health Research Ethics; University of Michigan Institutional Review Board; NHS Health Research Authority: North West - Haydock Research Ethics Committee.

Note that full information on the approval of the study protocol must also be provided in the manuscript.

Field-specific reporting

Please select the one below that is the best fit for your research. If you are not sure, read the appropriate sections before making your selection.

☒ Life sciences ☐ Behavioural & social sciences ☐ Ecological, evolutionary & environmental sciences

For a reference copy of the document with all sections, see nature.com/documents/nr-reporting-summary-flat.pdf

Life sciences study design

All studies must disclose on these points even when the disclosure is negative.

Sample size	Discovery project using maximum sample size available. Therefore, no sample-size calculation was performed.
Data exclusions	The following pre-established exclusions were applied to achieve a precise definition of Hashimoto's thyroiditis cases and controls for analysis.<br>For case definition:<br>- ICD codes for all other causes of hypothyroidism, thyroiditis, hyperthyroidism, or other thyroid related disorders<br>- Thyroid cancer (medical history / current)<br>- Post-partum thyroiditis<br>- Thyroid interfering medication (lithium or amiodarone)<br>- Medical history of thyroid surgery<br>- Medical history of radioactive iodine treatment

	For control definition, the following exclusions were applied:<br>- Individuals <18 years of age<br>- Any thyroid dysfunction
Replication	Replication of the GWAS results could not be conducted because no additional independent samples were available. However, we compared our GWAS association results with the results of a formerly published study.
Randomization	Samples were population based. The analyses were adjusted for age, sex and relevant study-specific covariates such as principal components for population stratification, study center and family-structure (e.g. by inclusion of the kinship matrix as a random effect) if applicable.
Blinding	Blinding was not relevant to this study design as only association analyses were conducted, i.e. no randomized controlled trials.

Reporting for specific materials, systems and methods

We require information from authors about some types of materials, experimental systems and methods used in many studies. Here, indicate whether each material, system or method listed is relevant to your study. If you are not sure if a list item applies to your research, read the appropriate section before selecting a response.

Materials & experimental systems

n/a	Involved in the study
☒	☐ Antibodies
☒	☐ Eukaryotic cell lines
☒	☐ Palaeontology and archaeology
☒	☐ Animals and other organisms
☒	☐ Clinical data
☒	☐ Dual use research of concern
☒	☐ Plants

Methods

n/a	Involved in the study
☒	☐ ChIP-seq
☒	☐ Flow cytometry
☒	☐ MRI-based neuroimaging

Plants

Seed stocks	*Report on the source of all seed stocks or other plant material used. If applicable, state the seed stock centre and catalogue number. If plant specimens were collected from the field, describe the collection location, date and sampling procedures.*
Novel plant genotypes	*Describe the methods by which all novel plant genotypes were produced. This includes those generated by transgenic approaches, gene editing, chemical/radiation-based mutagenesis and hybridization. For transgenic lines, describe the transformation method, the number of independent lines analyzed and the generation upon which experiments were performed. For gene-edited lines, describe the editor used, the endogenous sequence targeted for editing, the targeting guide RNA sequence (if applicable) and how the editor was applied.*
Authentication	*Describe any authentication procedures for each seed stock used or novel genotype generated. Describe any experiments used to assess the effect of a mutation and, where applicable, how potential secondary effects (e.g. second site T-DNA insertions, mosiacism, off-target gene editing) were examined.*
