# Supplementary material for: Multi-ancestry genome-wide association analyses provide insights into the genetic basis of Hashimoto’s thyroiditis
Source: Nat Genet. 2026 Jul 29;58(8):1855–65. doi: 10.1038/s41588-026-02704-w (PMC13447099; doi:10.1038/s41588-026-02704-w)
Supplement: Supplementary file 3 [file 41588_2026_2704_MOESM3_ESM.pdf]

nature portfolio

Peer Review File

Multi-ancestry genome-wide association analyses provide insights into the genetic basis of Hashimoto's thyroiditis

Corresponding Author: Professor Alexander Teumer

Version 0:

Decision Letter:

17th January 2025

Dear Alexander,

Your Article "Multi-ancestry GWAS on Hashimoto’s thyroiditis reveals new risk loci and insights into the interplay between the immune system and the thyroid" has been seen by two referees. You will see from their comments below that, while they find your work of potential interest, they have raised substantial concerns that must be addressed. In light of these comments, we cannot accept the manuscript for publication at this time, but we would be interested in considering a suitably revised version that addresses the referees' concerns.

We hope you will find the referees' comments useful as you decide how to proceed. If you wish to submit a substantially revised manuscript, please bear in mind that we will be reluctant to approach the referees again in the absence of major revisions.

To guide the scope of the revisions, the editors discuss the referee reports in detail within the team, including with the chief editor, with a view to identifying key priorities that should be addressed in revision, and sometimes overruling referee requests that are deemed beyond the scope of the current study. In this case, we ask that you carefully check and revise all locus annotations and nearest gene assignments for accuracy throughout and update downstream analyses and interpretations where needed, as highlighted by Reviewer #2. We also ask that you extend analyses where feasible to provide additional insights, as requested by Reviewer #1. We hope you will find this prioritized set of referee points to be useful when revising your study. Please do not hesitate to get in touch if you would like to discuss these issues further.

If you choose to revise your manuscript taking into account all reviewer and editor comments, please highlight all changes in the manuscript text file. At this stage, we will need you to upload a copy of the manuscript in MS Word .docx or similar editable format.

We are committed to providing a fair and constructive peer-review process. Do not hesitate to contact us if there are specific requests from the reviewers that you believe are technically impossible or unlikely to yield a meaningful outcome.

If revising your manuscript:

*1) Include a “Response to referees” document detailing, point-by-point, how you addressed each referee comment. If no action was taken to address a point, you must provide a compelling argument. This response will be sent back to the referees along with the revised manuscript.

*2) If you have not done so already, please begin to revise your manuscript so that it conforms to our Article format instructions, available here.
Refer also to any guidelines provided in this letter.

*3) Include a revised version of any required Reporting Summary: https://www.nature.com/documents/nr-reporting-summary.pdf
It will be available to referees (and, potentially, statisticians) to aid in their evaluation if the manuscript goes back for peer review.
A revised checklist is essential for re-review of the paper.

Please be aware of our guidelines on

digital image standards.

EXTENDED DATA FIGURES

When re-submitting your manuscript, please ensure that any supplementary figures and tables that are crucial to the manuscript's conclusions are converted into Extended Data figures and tables to increase visibility of these data. Extended Data figures and tables are online-only (present in the online PDF and full-text HTML versions of the paper), peer-reviewed display items that provide essential background to the article but are not included in the main article due to space constraints. A maximum of ten Extended Data display items (figures and tables) is permitted.

You may use the link below to submit your revised manuscript and related files:

Link Redacted

Note: This URL links to your confidential home page and associated information about manuscripts you may have submitted, or that you are reviewing for us. If you wish to forward this email to co-authors, please delete the link to your homepage.

If you wish to submit a suitably revised manuscript, we hope to receive it within 3 months. If you cannot send it within this time, please let us know. We will be happy to consider your revision so long as nothing similar has been accepted for publication at Nature Genetics or published elsewhere. Should your manuscript be substantially delayed without notifying us in advance and your article is eventually published, the received date would be that of the revised, not the original, version.

Please do not hesitate to contact me if you have any questions or would like to discuss the required revisions further.

Nature Genetics is committed to improving transparency in authorship. As part of our efforts in this direction, we are now requesting that all authors identified as 'corresponding author' on published papers create and link their Open Researcher and Contributor Identifier (ORCID) with their account on the Manuscript Tracking System (MTS), prior to acceptance. ORCID helps the scientific community achieve unambiguous attribution of all scholarly contributions. You can create and link your ORCID from the home page of the MTS by clicking on 'Modify my Springer Nature account'. For more information, please visit www.springernature.com/orcid.

Thank you for the opportunity to review your work.

Sincerely,
Kyle

Kyle Vogan, PhD
Senior Editor
Nature Genetics
https://orcid.org/0000-0001-9565-9665

Referee expertise:

Referee #1: Genetics, autoimmune diseases

Referee #2: Genetics, autoimmune diseases

Reviewers' Comments:

Reviewer #1 (Remarks to the Author):

This paper describes a very large, multi-ancestry GWAS on Hashimoto's thyroiditis (HT), identifying 58 novel associated variants and 28 novel associated loci. Six loci are specific to individuals of European ancestry, and variants at two loci show different effects between males and females. Numerous other analyses were conducted; for example, as expected, there are significant genetic correlations between HT and several other autoimmune diseases that are epidemiologically associated with HT, particularly RA, vitiligo, Sjogren syndrome, and celiac disease, as well as thyroid hormone levels and TPOAb status. This study offers important methodological improvements over previous HT GWAS, particularly careful trait phenotype definition and curation.

While this study is very well done and comprises a tour-de-force of modern GWAS analyses of state-of-art publicly available omics data, the results nevertheless are largely descriptive. No functional analyses were conducted, and it seems puzzling that several potential opportunities for more in-depth analyses were apparently not pursued that might have led to new insights. Overall, the results of the present study do not greatly advance understanding of the basic biology of HT, of its causation and triggering in genetically susceptible individuals, or of its possible prevention; treatment of HT is already quite

effective and inexpensive via hormone replacement. Accordingly, the impact of this study is evolutionary, not revolutionary.

Specific comments:

1. From the beginning (line 134), the authors promulgate a common misperception of autoimmune diseases. While a number of autoimmune diseases, including HT, exhibit strong female predominance, that is not the case for some other autoimmune diseases, such as type 1 diabetes, ankylosing spondylitis, and vitiligo.

2. This reviewer found it difficult to ascertain whether or not (and if so, how) cases and controls in the current multi-ancestry GWAS overlap subjects included in previous HT GWAS and, if there was overlap, how the results compared within those cohorts in the current study versus previous studies, potentially providing insight into how improved trait definition and curation in the present study affected the results. For example, did improved trait definition demonstrably improve power?

3. The results of this multi-ancestry GWAS suggest that, overall, the underlying biology of HT is fundamentally similar across populations. Do the ancestry-specific variants that were observed fit into that framework? Do any of the ancestry-specific variants point to possible regional environmental differences in HT triggering?

4. The observation of sex-specific susceptibility associations is of considerable potential interest and potential importance. To what extent do the observed sex-specific associations account for the observed female-predominance of HT? There seems to have been no formal analysis of this question.

Reviewer #2 (Remarks to the Author):

This manuscript details the results of a meta-analysis of GWAS of Hashimoto's thyroiditis that expands the known risk loci by 58 variants in 28 genes. They go on to functionally direct these regions using various bioinformatics tools to better understand the impact on disease. However, a significant problem in the gene annotations in Table 1 and Figure 2 bring into question the rest of the manuscript since much of these conclusions are based on the locations of these associated variants to protein coding genes.

1). Table 1 lists BLK on 1p36.22 with rs5746009. This SNP is located at this cytoband, but the nearest gene is TNFRSF1B. BLK is located at 8p23.1.

2). Table 1 lists IRF5 on 11q23.3 with rs11605461. This SNP is located at this cytoband, but the nearest gene is CADM1. IRF5 is located at 7q32.

3). While not detailed here, there are more errors in Table 1.

4). Figure 2 has many genes misannotated including those described in Table 1 comments above.

5). There are no details provided on how ancestry analyses were done that was included as covariate in the analyses.

Version 1:

Decision Letter:

3rd June 2025

Dear Alexander,

Your revised Article "Multi-ancestry GWAS on Hashimoto’s thyroiditis reveals new risk loci and insights into the interplay between the immune system and the thyroid" has been seen by the original referees. You will see from their comments below that, while they find the manuscript improved, they have requested a few additional revisions to improve clarity and add further context to the findings. We remain interested in the possibility of publishing your study in Nature Genetics, but we would like to see your response to these points in the form of a further revision before we make a final decision on publication.

We therefore invite you to further revise your manuscript taking into account all reviewer comments. Please highlight all changes in the manuscript text file. At this stage, we will need you to upload a copy of the manuscript in MS Word .docx or similar editable format.

We are committed to providing a fair and constructive peer-review process. Do not hesitate to contact us if there are specific requests from the reviewers that you believe are technically impossible or unlikely to yield a meaningful outcome.

When revising your manuscript:

*1) Include a “Response to referees” document detailing, point-by-point, how you addressed each referee comment. If no

action was taken to address a point, you must provide a compelling argument. This response will be sent back to the referees along with the revised manuscript.

*2) If you have not done so already, please begin to revise your manuscript so that it conforms to our Article format instructions, available
here.
Refer also to any guidelines provided in this letter.

*3) Include a revised version of any required Reporting Summary: https://www.nature.com/documents/nr-reporting-summary.pdf
It will be available to referees (and, potentially, statisticians) to aid in their evaluation if the manuscript goes back for peer review.
A revised checklist is essential for re-review of the paper.

Please be aware of our guidelines on digital image standards.

EXTENDED DATA FIGURES

When re-submitting your manuscript, please ensure that any supplementary figures and tables that are crucial to the manuscript's conclusions are converted into Extended Data figures and tables to increase visibility of these data. Extended Data figures and tables are online-only (present in the online PDF and full-text HTML versions of the paper), peer-reviewed display items that provide essential background to the article but are not included in the main article due to space constraints. A maximum of ten Extended Data display items (figures and tables) is permitted.

Please use the link below to submit your revised manuscript and related files:

Link Redacted

Note: This URL links to your confidential home page and associated information about manuscripts you may have submitted, or that you are reviewing for us. If you wish to forward this email to co-authors, please delete the link to your homepage.

We hope to receive your revised manuscript within 4-8 weeks. If you cannot send it within this time, please let us know.

Please do not hesitate to contact me if you have any questions or would like to discuss these revisions further.

Nature Genetics is committed to improving transparency in authorship. As part of our efforts in this direction, we are now requesting that all authors identified as 'corresponding author' on published papers create and link their Open Researcher and Contributor Identifier (ORCID) with their account on the Manuscript Tracking System (MTS), prior to acceptance. ORCID helps the scientific community achieve unambiguous attribution of all scholarly contributions. You can create and link your ORCID from the home page of the MTS by clicking on 'Modify my Springer Nature account'. For more information, please visit www.springernature.com/orcid.

We look forward to seeing the revised manuscript and thank you for the opportunity to review your work.

Sincerely,
Kyle

Kyle Vogan, PhD
Senior Editor
Nature Genetics
https://orcid.org/0000-0001-9565-9665

Referee expertise:

Referee #1: Genetics, autoimmune diseases

Referee #2: Genetics, autoimmune diseases

Reviewers' Comments:

Reviewer #1 (Remarks to the Author):

The authors have addressed my concerns, and the revised manuscript is far more tightly reasoned, justified, and written than was the original version, and thus has been much improved overall. While the impact remains evolutionary rather than

revolutionary, the results constitute a significant set of data that advance overall knowledge and understanding of HT. Nevertheless, this reviewer sees no real importance to the results of the Drug Target Analysis (except to say that this was done), and this section can thus be deleted without negatively affecting the manuscript. Further, there appears to have been little editing of the Discussion, which remains quite 'wordy'. The Discussion would benefit from condensation to highlight the most important results and conclusions.

That said, this reviewer is dismayed by the observations of numerous errors of annotation in the previous version. I have not taken the time to go through these, but it is strongly recommended that a senior author do so before the results are published or submitted to online databases.

Similarly, numerous subtle errors of English and typos remain, even in the revised manuscript, and use of commas is haphazard at best. It would be well for someone to go through the manuscript carefully prior to publication.

Reviewer #2 (Remarks to the Author):

The authors have corrected the issues with the annotation of the GWAS data and have addressed most of the comments from the other reviewer. However, there are a few outstanding issues that need further attention.

1. Line 153, Please specify what ancestries were used in multi-ancestry analysis as it is just briefly in Discussion line 412 specify. Please provide the number of cases and controls included with each of ancestry from each biobank source that was meta-analyzed. In Supplementary Table 1 for non-European, you have 997 cases, 25,643 controls in All of Us, 1,597 cases, 172,352 controls in Biobank Japan, 3,610 cases and 72,719 controls in UKBB, please specify which ancestry they are and add it to Figure 1.

Line 155 and Methods Line 535, and Supplementary Figure 2 specified ICD9/10 inclusion criteria for autoimmune hypothyroidism and unspecified hypothyroidism, excluding all other thyroid disease codes, what are your exclusion criteria for controls? These results are not compared to other papers on hypothyroidism, and some of presented associations are shown in GWASs on hypothyroidism [1,2].

Could you please specify how your novel loci are specific to autoimmune hypothyroidism, especially compared to hypothyroidism? Or why not compare the results for the results of the GWAS presented here with the excluded codes to prove these are specific for autoimmune hypothyroidism? Figuerêdo et al. [3] performed GWAS on 58,783 cases with summary statistics from EstBB, FinnGen, UKBB (ICD-10/9: E00, E01, E02, E03, E089, 244, 243, overlapping with your criteria 244.9, E03.9) and they found a lot of novel association shown in this paper (e.g. rs7520586, rs79723498).

[1] Verma A, Huffman JE, Rodriguez A, Conery M, Liu M, Ho YL, Kim Y, Heise DA, Guare L, Panickan VA, Garcon H, Linares F, Costa L, Goethert I, Tipton R, Honerlaw J, Davies L, Whitbourne S, Cohen J, Posner DC, Sangar R, Murray M, Wang X, Dochtermann DR, Devineni P, Shi Y, Nandi TN, Assimes TL, Brunette CA, Carroll RJ, Clifford R, Duvall S, Gelernter J, Hung A, Iyengar SK, Joseph J, Kember R, Kranzler H, Kripke CM, Levey D, Luoh SW, Merritt VC, Overstreet C, Deak JD, Grant SFA, Polimanti R, Roussos P, Shakt G, Sun YV, Tsao N, Venkatesh S, Voloudakis G, Justice [3] A, Begoli E, Ramoni R, Tourassi G, Pyarajan S, Tsao P, O'Donnell CJ, Muralidhar S, Moser J, Casas JP, Bick AG, Zhou W, Cai T, Voight BF, Cho K, Gaziano JM, Madduri RK, Damrauer S, Liao KP. Diversity and scale: Genetic architecture of 2068 traits in the VA Million Veteran Program. Science. 2024 Jul 19;385(6706):eadj1182. doi: 10.1126/science.adj1182. Epub 2024 Jul 19. PMID: 39024449.

[2] Kichaev G, Bhatia G, Loh PR, Gazal S, Burch K, Freund MK, Schoech A, Pasaniuc B, Price AL. Leveraging Polygenic Functional Enrichment to Improve GWAS Power. Am J Hum Genet. 2019 Jan 3;104(1):65-75. doi: 10.1016/j.ajhg.2018.11.008. Epub 2018 Dec 27. PMID: 30595370; PMCID: PMC6323418.

[3] Figuerêdo J, Krebs K, Pujol-Gualdo N, Haller T, Võsa U, Volke V, Laisk T, Mägi R; Estonian Biobank Research Team; Health Informatics Research Team. Uncovering the shared genetic components of thyroid disorders and reproductive health. Eur J Endocrinol. 2024 Aug 5;191(2):211-222. doi: 10.1093/ejendo/lvae094. PMID: 39067062.

2. In Line 218, the authors state East Asian non-European. Is this in reference to the Japanese Biobank data or are there others that were meta-analyzed from other biobanks? If an East-Asian GWAS was done combining more than one cohort, please provide the specific details in the methods.

3. Line 239, Pfemale is not given for the male specific variant, rs10068965, but in the next sentence both Pmale and Pfemale is given for female specific variant rs7695810. Please provide Pfemale for rs10068965.

4. Line 411-412, The number of individuals is inflated by controls from biobanks. Since the exact numbers are provided right after, it would be best to remove the 1.2 million as this is mostly controls.

5. Line 578, Please specify the threshold used to test for between study heterogeneity. Variants should be excluded that show >50% heterogeneity. Also, please calculate the Conchran's Q values and exclude those with p<0.05.

The Combination of Estimates from Different Experiments on JSTOR

William G. Cochran, The Combination of Estimates from Different Experiments, Biometrics, Vol. 10, No. 1 (Mar., 1954), pp. 101-129

6. Figure 1 – Regeneron DiscovEHR controls have comma in wrong place, please add the numbers of non-European cases, if possible split it by ancestry in your analysis.

7. Figure 3 and Figure 4, the y-axis shows -log(10), is labeled as log(10), the title of plot is Hypothyroidism, not Hashimoto's thyroiditis. In Hypothyroidism, some of those novel loci were previously descripted in other published manuscripts listed above.

8. Line 970, Figure 3 legend describe males (top) females (bottom), but it looks like it is on reverse on Figure 3.

9. Line 977, Please add locus plots and LD plots for female/male variant of CAPZB.

10. Table 1, please add a column for the nearest gene, as some of this association is known in literature by the nearest gene.

11. The Manhattan plots as shown with the gene annotations will be very hard to read in publication.

Version 2:

Decision Letter:

25th September 2025

Dear Alexander,

Your revised Article "Multi-ancestry GWAS on Hashimoto's thyroiditis reveals new risk loci and insights into the interplay between the immune system and the thyroid" has been seen again by Reviewer #2, and as you will see from the comments below, Reviewer #2 has a few ongoing concerns. As before, we remain interested in the possibility of publishing your study in Nature Genetics, but we would like to consider your response to these remaining concerns in the form of a further revision before we make a final decision on publication.

In particular, we ask that you provide further details on the sample sizes of each ancestry subgroup represented in the two multi-ancestry cohorts (All of Us and UK Biobank), clarify how ancestry was accounted for when performing genome-wide association analyses within these cohorts, and further qualify claims for loci showing evidence for effect size heterogeneity. We hope you will find this prioritized set of referee points to be useful when revising your study. Please do not hesitate to get in touch if you would like to discuss these issues further.

We therefore invite you to revise your manuscript taking into account all reviewer and editor comments. Please highlight all changes in the manuscript text file. At this stage, we will need you to upload a copy of the manuscript in MS Word .docx or similar editable format.

We are committed to providing a fair and constructive peer-review process. Do not hesitate to contact us if there are specific requests from the reviewers that you believe are technically impossible or unlikely to yield a meaningful outcome.

When revising your manuscript:

*1) Include a "Response to referees" document detailing, point-by-point, how you addressed each referee comment. If no action was taken to address a point, you must provide a compelling argument. This response will be sent back to the referees along with the revised manuscript.

*2) If you have not done so already, please begin to revise your manuscript so that it conforms to our Article format instructions, available
here.
Refer also to any guidelines provided in this letter.

*3) Include a revised version of any required Reporting Summary: https://www.nature.com/documents/nr-reporting-summary.pdf
It will be available to referees (and, potentially, statisticians) to aid in their evaluation if the manuscript goes back for peer review.
A revised checklist is essential for re-review of the paper.

Please be aware of our guidelines on digital image standards.

EXTENDED DATA FIGURES

When re-submitting your manuscript, please ensure that any supplementary figures and tables that are crucial to the manuscript's conclusions are converted into Extended Data figures and tables to increase visibility of these data. Extended Data figures and tables are online-only (present in the online PDF and full-text HTML versions of the paper), peer-reviewed display items that provide essential background to the article but are not included in the main article due to space constraints. A maximum of ten Extended Data display items (figures and tables) is permitted.

Please use the link below to submit your revised manuscript and related files:

Link Redacted

Note: This URL links to your confidential home page and associated information about manuscripts you may have submitted, or that you are reviewing for us. If you wish to forward this email to co-authors, please delete the link to your homepage.

We again hope to receive your revised manuscript within 4-8 weeks. If you cannot send it within this time, please let us know.

Please do not hesitate to contact me if you have any questions or would like to discuss these revisions further.

Nature Genetics is committed to improving transparency in authorship. As part of our efforts in this direction, we are now requesting that all authors identified as 'corresponding author' on published papers create and link their Open Researcher and Contributor Identifier (ORCID) with their account on the Manuscript Tracking System (MTS), prior to acceptance. ORCID helps the scientific community achieve unambiguous attribution of all scholarly contributions. You can create and link your ORCID from the home page of the MTS by clicking on 'Modify my Springer Nature account'. For more information, please visit www.springernature.com/orcid.

We look forward to seeing the revised manuscript and thank you for the opportunity to review your work.

Sincerely,
Kyle

Kyle Vogan, PhD
Senior Editor
Nature Genetics
https://orcid.org/0000-0001-9565-9665

Referee expertise:

Referee #2: Genetics, autoimmune diseases

Reviewers' Comments:

Reviewer #2 (Remarks to the Author):

While the authors are working to try to resolve some the comments, there are some points that still need attention.

1). It is commonplace to report the numbers of subjects in ancestry groups in GWAS. While it is likely these numbers are low and you likely do not want to highlight this, it is important to acknowledge the ancestry driving the findings of this paper. Thus, please report the ancestry (mixed ancestry is okay answer too) so that the sample size of the non-European component can be assessed.

2). It still is not clear how, if you are not defining ancestry, you are able to correct for ancestry enough to do a study including multiple ancestries. Best practices indicate meta-analysis of summary stats is the best approach, not merging of genotype data from multiple ancestries.

3). Of the risk loci reported, heterogeneity has been observed in many of these loci, such as IRF5 and BLK. The I2 of 75% is too high and these threshold of >50% (Moderate heterogeneity) to be considered.

Version 3:

Decision Letter:

Our ref: NG-A66836R2

18th February 2026

Dear Alexander,

Thank you for submitting your revised manuscript "Multi-ancestry GWAS on Hashimoto's thyroiditis reveals new risk loci and insights into the interplay between the immune system and the thyroid" (NG-A66836R2). In light of your responses and clarifications, we will be happy in principle to publish your study in Nature Genetics as an Article pending final revisions to comply with our editorial and formatting guidelines.

We are now performing detailed checks on your paper, and we will send you a checklist detailing our editorial and formatting requirements soon. Please do not upload the final materials or make any revisions until you receive this additional information from us.

Thank you again for your interest in Nature Genetics. Please do not hesitate to contact me if you have any questions.

Sincerely,
Kyle

Kyle Vogan, PhD
Senior Editor
Nature Genetics
https://orcid.org/0000-0001-9565-9665

Open Access This Peer Review File is licensed under a Creative Commons Attribution 4.0 International License, which permits use, sharing, adaptation, distribution and reproduction in any medium or format, as long as you give appropriate credit to the original author(s) and the source, provide a link to the Creative Commons license, and indicate if changes were made.
In cases where reviewers are anonymous, credit should be given to 'Anonymous Referee' and the source.
The images or other third party material in this Peer Review File are included in the article's Creative Commons license, unless indicated otherwise in a credit line to the material. If material is not included in the article's Creative Commons license and your intended use is not permitted by statutory regulation or exceeds the permitted use, you will need to obtain permission directly from the copyright holder.
To view a copy of this license, visit https://creativecommons.org/licenses/by/4.0/

Response to referees' comments manuscript "Multi-ancestry GWAS on Hashimoto's thyroiditis reveals new risk loci and insights into the interplay between the immune system and the thyroid " (NG-A66836R)

We thank the referees again for thoroughly checking our manuscript, and for bringing up valid and important points to our attention.

Based on the suggestion of reviewer 1, we moved the Drug Target Analysis section into the supplemental material. As this part might still contain valuable information for readers, we were reluctant to remove it entirely from the manuscript but will do so if needed.

Modifications in the manuscript are highlighted in track-mode.

Reviewer 1

The authors have addressed my concerns, and the revised manuscript is far more tightly reasoned, justified, and written than was the original version, and thus has been much improved overall. While the impact remains evolutionary rather than revolutionary, the results constitute a significant set of data that advance overall knowledge and understanding of HT. Nevertheless, this reviewer sees no real importance to the results of the Drug Target Analysis (except to say that this was done), and this section can thus be deleted without negatively affecting the manuscript. Further, there appears to have been little editing of the Discussion, which remains quite 'wordy'. The Discussion would benefit from condensation to highlight the most important results and conclusions.

We agree and have now edited the discussion extensively, making it less wordy.

That said, this reviewer is dismayed by the observations of numerous errors of annotation in the previous version. I have not taken the time to go through these, but it is strongly recommended that a senior author do so before the results are published or submitted to online databases.

We apologize again for this annotation mismatch that occurred when adding the Open Targets gene names to the supplementary GWAS results table. Unfortunately, these tables provided the basis for all subsequent annotations and plots. However, besides the assigned Open Targets gene annotations, the results were not affected by this error which has been corrected after the first revision as also confirmed by reviewer 2.

Similarly, numerous subtle errors of English and typos remain, even in the revised manuscript, and use of commas is haphazard at best. It would be well for someone to go through the manuscript carefully prior to publication.

We went again through the manuscript and corrected several typos and commas.

Reviewer 2

The authors have corrected the issues with the annotation of the GWAS data and have addressed most of the comments from the other reviewer. However, there are a few outstanding issues that need further attention.

1. Line 153, Please specify what ancestries were used in multi-ancestry analysis as it is just briefly in Discussion line 412 specify. Please provide the number of cases and controls included with each of ancestry from each biobank source that was meta-analyzed. In Supplementary Table 1 for non-European, you have 997 cases, 25,643 controls in All of Us, 1,597 cases, 172,352 controls in Biobank Japan, 3,610 cases and 72,719 controls in UKBB, please specify which ancestry they are and add it to Figure 1.

We value this remark and added the corresponding ancestry information to the Figure 1 and its description. Categorizing individuals into genetic ancestry groups is generally difficult and remains arbitrary, as for example emphasized in reference [1] below. As we conducted solely a multi-ancestry and a European ancestry GWAS meta-analysis without assessing further ancestry groups, we would like to stay on the safe side and refrain from reporting numbers of cases of specific non-European ancestries included in our multi-ancestry GWAS.

Line 155 and Methods Line 535, and Supplementary Figure 2 specified ICD9/10 inclusion criteria for autoimmune hypothyroidism and unspecified hypothyroidism, excluding all other thyroid disease codes, what are your exclusion criteria for controls?

Thank you for bringing up this issue. Individuals without thyroid dysfunction and over the age of 18 years are considered as controls. We added the detailed control definition from our GWAS analysis plan now to Supplementary Table 2, and provide that information in line 155.

These results are not compared to other papers on hypothyroidism, and some of presented associations are shown in GWASs on hypothyroidism [1,2].

In the initial submission of our manuscript, we compared our Hashimoto's thyroiditis GWAS results to the meta-analysis of Mathieu *et al.* (2022) totaling up to 51,194 European ancestry hypothyroidism cases obtained from the UKBB and FinnGen, and replicated in 17,002 cases of the EstBB. To the best of our knowledge, this study represented the largest hypothyroidism GWAS with the best matching trait definition published at the time we finalized our project. The meta-analysis of Mathieu *et al.* implemented a more general case definition of hypothyroidism (i.e. individuals taking thyroid medication). Using the more specific case definition, we revealed 58 novel genetic variants and 28 novel loci (Supplementary Table 3) while our number of cases was slightly smaller.

However, to stay conservative with reporting the number of new findings, we now also took the publications of Verma *et al.* [2] and Figuerêdo *et al.* [3] into account. The latter publication used a broader trait definition including also 'congenital iodine-deficiency syndrome and hypothyroidism', 'iodine-deficiency-related thyroid disorders and allied conditions', and 'iodine-deficiency-related thyroid disorders'. Nevertheless, our GWAS meta-analysis still revealed 45 novel variants in 19 previously undiscovered loci (Supplementary Table 3).

Of note, is was neither possible to retrieve the definition of hypothyroidism nor the number of cases used in the UKBB GWAS of Kichaev *et al.* from the corresponding publication [2]. Taking additionally into account that the number of revealed hypothyroidism associations was lower compared to meta-analyses

that added additional cohorts to the UKBB dataset (i.e. Mathieu *et al.*, Figuerêdo *et al.*), we do not think that reference [2] provides a dataset that allows a meaningful comparison for our Hashimoto's thyroiditis GWAS results.

Could you please specify how your novel loci are specific to autoimmune hypothyroidism, especially compared to hypothyroidism? Or why not compare the results for the results of the GWAS presented here with the excluded codes to prove these are specific for autoimmune hypothyroidism? Figuerêdo et al. [3] performed GWAS on 58,783 cases with summary statistics from EstBB, FinnGen, UKBB (ICD-10/9: E00, E01, E02, E03, E089, 244, 243, overlapping with your criteria 244.9, E03.9) and they found a lot of novel association shown in this paper (e.g. rs7520586, rs79723498).

We thank this reviewer for bringing up this important aspect, which is similar to the question 2 raised by reviewer 1 in the former revision round. Taking into account the partial overlap in the selection criteria for cases and controls applied in our Hashimoto's GWAS with the trait definition applied in the published GWAS meta-analyses, this question cannot be fully answered. However, to provide further insights whether our hits might be specific for Hashimoto's hypothyroidism, we now show in Supplementary Table 3 the overlap of our results with the outcome of the broad hypothyroidism case assessment from Figuerêdo [3] as suggested by the reviewer. In addition, we compared the effect sizes of our GWAS meta-analysis with the effect sizes revealed in reference [3] in Reviewer Figure 1. The effect sizes (after aligning to the trait increasing allele from our GWAS) of the 146 displayed variants were on average significantly larger in our Hashimoto's meta-analysis compared to the hypothyroidism effects available in the Figuerêdo dataset (diff = 0.006, p = 0.001). This trend could still be observed when limiting this analysis to the known variants reducing potential overestimation by our new findings (diff = 0.002, p = 0.30). In addition, of the 141 significant loci associated with general hypothyroidism in [3], 49 did not appear among the loci identified in our Hashimoto's GWAS meta-analysis.

Our case and control definition was specifically chosen to assess autoimmune hypothyroidism while separating the trait definition from other types of hypothyroidism and autoimmune thyroid diseases like Graves' disease. Therefore, our hits represent associations with Hashimoto's thyroiditis although they may have pleiotropic effects on other thyroid disorders.

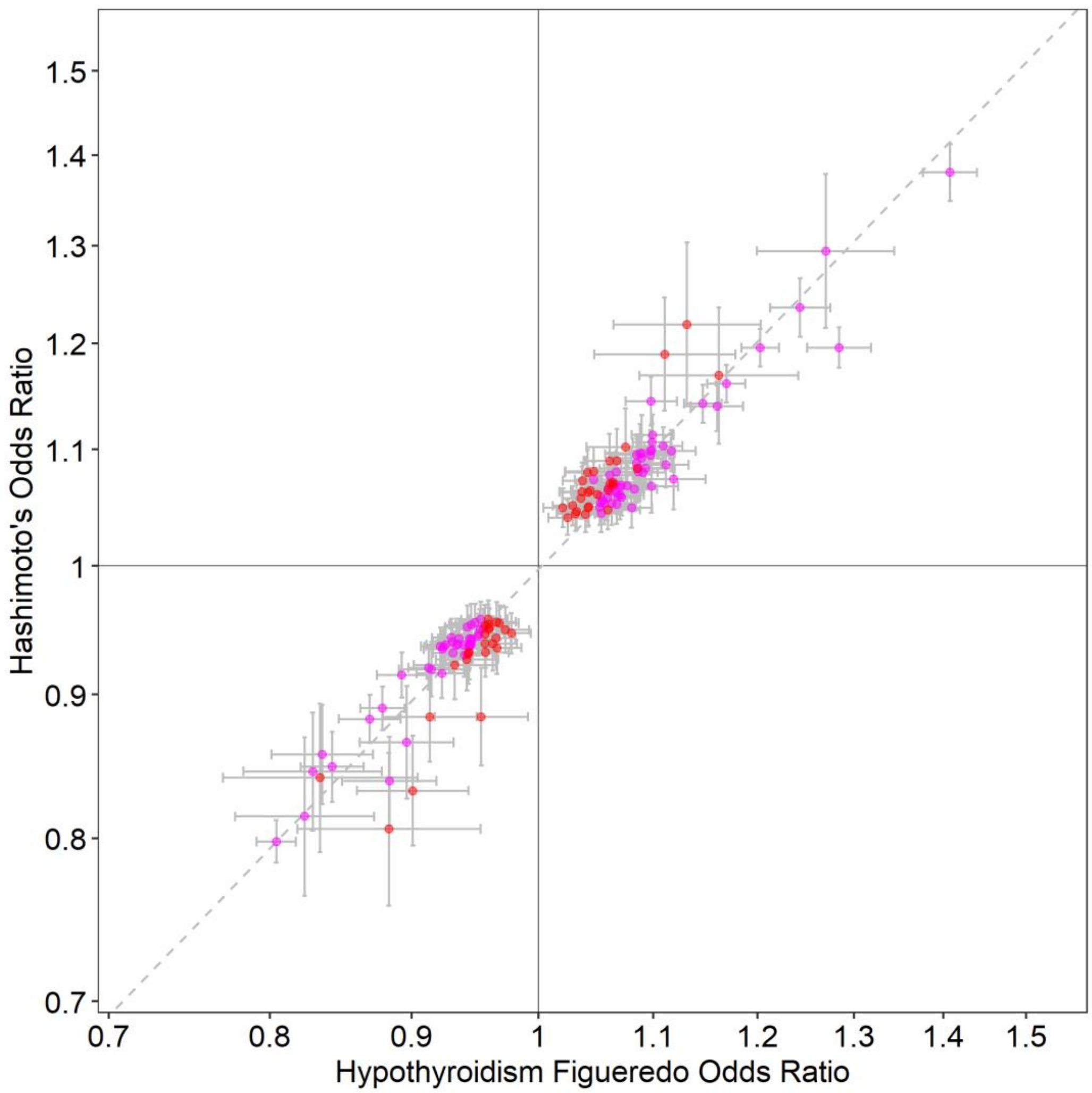

Reviewer Figure 1: Comparison of the effect sizes of our significant Hashimoto's thyroiditis multi-ancestry GWAS meta-analysis results (y-axis) with the corresponding effect sizes in the hypothyroidism GWAS meta-analysis of Figuerêdo *et al.* [3] (x-axis). Of our 155 associations of the multi-ancestry GWAS meta-analysis, 146 were available in the Figuerêdo GWAS and displayed. Genetic associations that were specific to our GWAS only and in both datasets are displayed in red (n=52) and purple (n=94), respectively. The whiskers represent the 95% confidence intervals of the corresponding odds ratios.

[1] Verma A, Huffman JE, Rodriguez A, Conery M, Liu M, Ho YL, Kim Y, Heise DA, Guare L, Panickan VA, Garcon H, Linares F, Costa L, Goethert I, Tipton R, Honerlaw J, Davies L, Whitbourne S, Cohen J, Posner DC, Sangar R, Murray M, Wang X, Dochtermann DR, Devineni P, Shi Y, Nandi TN, Assimes TL, Brunette CA, Carroll RJ, Clifford R, Duvall S, Gelernter J, Hung A, Iyengar SK, Joseph J, Kember R, Kranzler H, Kripke CM, Levey D, Luoh SW, Merritt VC, Overstreet C, Deak JD, Grant SFA, Polimanti R, Roussos P, Shakt G, Sun YV, Tsao N, Venkatesh S, Voloudakis G, Justice [3] A, Begoli E, Ramoni R, Tourassi G, Pyarajan S, Tsao P, O'Donnell CJ, Muralidhar S, Moser J, Casas JP, Bick AG, Zhou W, Cai T, Voight BF, Cho K, Gaziano JM, Madduri RK, Damrauer S, Liao KP. Diversity and scale: Genetic architecture of 2068 traits in the VA Million Veteran Program. Science. 2024 Jul 19;385(6706):eadj1182. doi: 10.1126/science.adj1182. Epub 2024 Jul 19. PMID: 39024449.

[2] Kichaev G, Bhatia G, Loh PR, Gazal S, Burch K, Freund MK, Schoech A, Pasaniuc B, Price AL. Leveraging Polygenic Functional Enrichment to Improve GWAS Power. Am J Hum Genet. 2019 Jan 3;104(1):65-75. doi: 10.1016/j.ajhg.2018.11.008. Epub 2018 Dec 27. PMID: 30595370; PMCID: PMC6323418.

[3] Figuerêdo J, Krebs K, Pujol-Gualdo N, Haller T, Võsa U, Volke V, Laisk T, Mägi R; Estonian Biobank Research Team; Health Informatics Research Team. Uncovering the shared genetic components of thyroid disorders and reproductive health. Eur J Endocrinol. 2024 Aug 5;191(2):211-222. doi: 10.1093/ejendo/lvae094. PMID: 39067062.

2. In Line 218, the authors state East Asian non-European. Is this in reference to the Japanese Biobank data or are there others that were meta-analyzed from other biobanks? If an East-Asian GWAS was done combining more than one cohort, please provide the specific details in the methods.

We indeed referred to a lookup in the Japanese Biobank GWAS as we did not run dedicated non-European ancestry specific GWAS (and meta-analyses) for reasons of statistical power. The Biobank Japan includes 100% East Asian ancestry individuals in their GWAS dataset. However, our multi-ancestry GWAS and meta-analysis included additional non-European (and East-Asian) individuals and was part of the comparison of the effect sizes of European ancestry specific results among other ancestries.

3. Line 239, Pfemale is not given for the male specific variant, rs10068965, but in the next sentence both Pmale and Pfemale is given for female specific variant rs7695810. Please provide Pfemale for rs10068965.

The corresponding p-values of both the female and sex-combined stratum were added now.

4. Line 411-412, The number of individuals is inflated by controls from biobanks. Since the exact numbers are provided right after, it would be best to remove the 1.2 million as this is mostly controls.

We removed the 1.2 million accordingly from the respective Discussion and Introduction sections.

5. Line 578, Please specify the threshold used to test for between study heterogeneity. Variants should be excluded that show >50% heterogeneity. Also, please calculate the Conchran's Q values and exclude those with p<0.05. (The Combination of Estimates from Different Experiments on JSTOR. William G. Cochran, The Combination of Estimates from Different Experiments, Biometrics, Vol. 10, No. 1 (Mar., 1954), pp. 101-129)

We calculated the I^2 measure to quantify the heterogeneity between the GWAS studies, and provide this information in the corresponding Supplementary Tables 3,5,7 and 8 to the reader. The I^2 value overcomes several limitations of the Cochran's Q test statistics for detecting heterogeneity. The significance of the Q statistics is dependent on the number of studies and the sample size of the studies included in the meta-analysis (Higgins *et al.*, BMJ 2003, PMID: 12958120). Furthermore, heterogeneous GWAS results may also be informative and represent true positive associations. Our significant associations with high heterogeneity (I^2>75% according to Higgins *et al.*) are located in the HLA region and/or have ancestry related heterogeneity, which is plausible. Thus, we did not apply (also in concordance with many of published GWAS meta-analyses, including Figuerêdo *et al.* [3]) any heterogeneity-based variant filtering to prevent removing informative results. However, as suggested by the Reviewer we added the corresponding p-values of the Cochran's Q test statistics to these Supplementary Tables.

6. Figure 1 – Regeneron DiscovEHR controls have comma in wrong place, please add the numbers of non-European cases, if possible split it by ancestry in your analysis.

We corrected the comma in Figure 1. The Regeneron DiscovEHR dataset included in this project included solely individuals of European ancestry as already indicated in the Figure 1 and Supplementary Table 1.

7. Figure 3 and Figure 4, the y-axis shows -log(10), is labeled as log(10), the title of plot is Hypothyroidism, not Hashimoto's thyroiditis. In Hypothyroidism, some of those novel loci were previously descripted in other published manuscripts listed above.

The $-\log_{10}$ y-axis labels are plotted correctly now, we changed all plot titles to Hashimoto's thyroiditis, and limited the labels to the 20 loci that were not published previously for a hypothyroidism trait according to answer 1.

8. Line 970, Figure 3 legend describe males (top) females (bottom), but it looks like it is on reverse on Figure 3.

Thank you for catching this issue. We corrected the legend accordingly.

9. Line 977, Please add locus plots and LD plots for female/male variant of CAPZB.

We provide the two regional association plots including LD information as new Supplementary Figure 2.

10. Table 1, please add a column for the nearest gene, as some of this association is known in literature by the nearest gene.

This information was added to the Table 1, which is limited now also to the 19 loci that were not published previously for a hypothyroidism trait.

11. The Manhattan plots as shown with the gene annotations will be very hard to read in publication.

We modified the double Manhattan plots in Figure 3 and Extended Data Figure 2 accordingly.

Response to referees' comments manuscript "Multi-ancestry GWAS on Hashimoto's thyroiditis reveals new risk loci and insights into the interplay between the immune system and the thyroid " (NG-A66836R1)

We thank the reviewer again for thoroughly checking our manuscript. We are happy to address the remaining concerns which are provided in a point-by-point response below.

Modifications in the manuscript are highlighted in track-mode.

Reviewer 2

1). It is commonplace to report the numbers of subjects in ancestry groups in GWAS. While it is likely these numbers are low and you likely do not want to highlight this, it is important to acknowledge the ancestry driving the findings of this paper. Thus, please report the ancestry (mixed ancestry is okay answer too) so that the sample size of the non-European component can be assessed.

We agree with the Reviewer that it is important to get am impression which ancestries are driving our GWAS findings. We added the exact number of cases and controls of the genetically inferred ancestry groups included in the two mixed-ancestry cohorts (All Of Us and the UK Biobank) to Supplementary Table 1.

Furthermore, we added a corresponding summary to the Results section (line 148): "The individuals included were predominantly but not exclusively of genetically inferred European ancestry (European: 75%, East Asian: 16%, African: 2%, South Asian and Middle Eastern: 1%, admixed/unknown: 6%) (Supplementary Table 1)."

We would like to reiterate that categorizing individuals into genetic ancestry groups is generally difficult and remains somewhat arbitrary. The precision of the assignment of individuals to the reported ancestry groups has limitations and the proportional breakdown is therefore intended as a general overview.

2). It still is not clear how, if you are not defining ancestry, you are able to correct for ancestry enough to do a study including multiple ancestries. Best practices indicate meta-analysis of summary stats is the best approach, not merging of genotype data from multiple ancestries.

We meta-analyzed the summary statistics of cohort-specific GWAS analyses. Within cohorts, genetic principal components (PCs) were added as covariates in the individual GWAS to correct for population substructure including structure introduced by differences in genetic ancestries. In the UK Biobank, we included the first 15 PCs. These PCs were calculated on the whole UK Biobank sample to correctly map the ancestries and population structure of this dataset (see Bycroft *et al.*)[1].

As stated in that referenced publication (Supplement section 3.3), "the PCA we conducted serves two purposes: […]; and to assist the research community by computing a metric that is widely used as an indicator of genetic ancestry (complementary to self-reported ethnicity), and is widely used as a method for assessing and potentially controlling for population structure in GWAS[2], [3]."

To verify that the UK Biobank GWAS results are unaffected by including all 40 pre-calculated PCs instead of the first 15 PCs we used in the multi-ancestry meta-analysis, we conducted a sensitivity GWAS analysis. The results show that both the effect sizes and p-values are virtually the same (Reviewer Figure 1).

The same approach using PCs was also applied in the All Of Us study, thereby including 10 PCs.

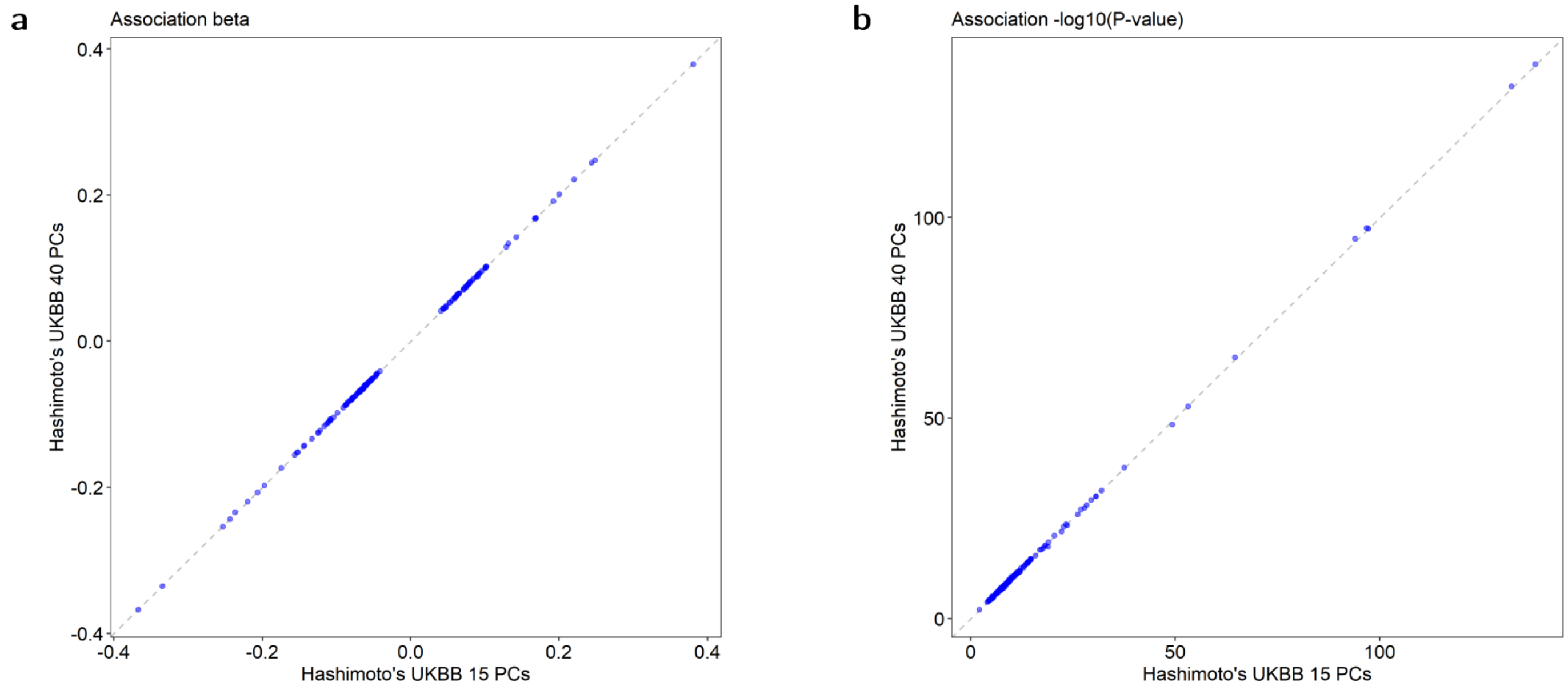

Reviewer Figure 1: Comparison of the effect sizes (a) and $-\log_{10}$ p-values (b) of the 155 significant Hashimoto's GWAS meta-analysis variants in the complete UK Biobank sample adjusting for 15 (x-axis) and 40 (y-axis) genetic principal components in the association model. The correlation coefficient equals 1 in both panels.

Regarding the proposed meta-analysis of ancestry-stratum summary statistics, a recent paper of Dias *et al.* using large-scale simulations and real data of both All of Us and UK Biobank as examples, demonstrated that pooled analysis generally exhibits better statistical power than an ancestry-specific analysis followed by cross-ancestry meta-analysis while effectively adjusting for population stratification[4]. They also elucidate via a theoretical framework that as with increasing allele-frequency divergence across ancestral groups, meta-analysis becomes less efficient compared to pooled analysis. Although both approaches are generally valid, the final choice of using ancestry-combined GWAS or ancestry-stratified GWAS per cohort likely depends on several factors including the specific research question and available sample size. We furthermore agree that many published studies stratified cohorts by ancestry when running the GWAS, but there are also recent studies that conducted ancestry combined GWAS using PCs including data from the UK Biobank and US cohorts[5], [6].

3). Of the risk loci reported, heterogeneity has been observed in many of these loci, such as IRF5 and BLK. The I2 of 75% is too high and these threshold of >50% (Moderate heterogeneity) to be considered.

A high level of heterogeneity in a meta-analysis as indicated by high I^2 values can be attributed to several phenomena, including true effect modifications which may reflect biologically interesting findings, as exemplified for the *UMOD/PDILT* locus, a well-known kidney disease locus where genetic effect size is modified by age [7]. In our analysis, some of the findings with the highest I^2 values include already published and validated associations in the HLA region that is known for its heterogeneity (rs9272426,

rs9273007, rs11754075, rs3130552). There are additional heterogeneous loci in other genetic regions, where several of them were well established associations and replicated in our study. Such observed high I^2 values are also found in other hypothyroidism GWAS like in Figuerêdo *et al.*[8] (I^2>80% in Table S3). Thus, we strongly believe that also GWAS results with high heterogeneity may be important and should be published along with their respective I^2 values, so that readers can interpret and validate the locus in light of this information. Of note, the *IRF5* and *BLK* loci mentioned by the Reviewer represent homogeneous associations (I^2=0) in our GWAS meta-analyses.

References:

[1] C. Bycroft *et al.*, “The UK Biobank resource with deep phenotyping and genomic data,” *Nat. 2018 5627726*, vol. 562, no. 7726, pp. 203–209, Oct. 2018, doi: 10.1038/s41586-018-0579-z.

[2] D. J. Balding, “A tutorial on statistical methods for population association studies,” *Nat. Rev. Genet.*, vol. 7, no. 10, pp. 781–791, Oct. 2006, doi: 10.1038/nrg1916.

[3] A. L. Price, N. J. Patterson, R. M. Plenge, M. E. Weinblatt, N. A. Shadick, and D. Reich, “Principal components analysis corrects for stratification in genome-wide association studies.,” *Nat Genet*, vol. 38, no. 8, pp. 904–909, Aug. 2006, doi: 10.1038/ng1847.

[4] J.-A. Dias *et al.*, “Evaluating multi-ancestry genome-wide association methods: Statistical power, population structure, and practical implications,” *Am. J. Hum. Genet.*, vol. 112, no. 10, pp. 2493–2508, Oct. 2025, doi: 10.1016/j.ajhg.2025.08.006.

[5] J. D. S. Willett *et al.*, “Identification of 16 novel Alzheimer’s disease loci using multi-ancestry meta-analyses.,” *Alzheimers. Dement.*, vol. 21, no. 2, p. e14592, Feb. 2025, doi: 10.1002/alz.14592.

[6] X. Zhang *et al.*, “Multi-ancestry whole genome sequencing analysis of lean body mass.,” *Genome Biol.*, vol. 26, no. 1, p. 106, Apr. 2025, doi: 10.1186/s13059-025-03520-x.

[7] M. Wuttke *et al.*, “A catalog of genetic loci associated with kidney function from analyses of a million individuals.,” *Nat. Genet.*, vol. 51, no. 6, pp. 957–972, Jun. 2019, doi: 10.1038/s41588-019-0407-x.

[8] J. Figuerêdo *et al.*, “Uncovering the shared genetic components of thyroid disorders and reproductive health.,” *Eur. J. Endocrinol.*, vol. 191, no. 2, pp. 211–222, Aug. 2024, doi: 10.1093/ejendo/lvae094.
